# A low-cost robotic hand prosthesis with apparent haptic sense controlled by electroencephalographic signals

**DOI:** 10.1016/j.ohx.2023.e00439

**Published:** 2023-06-02

**Authors:** Diego Ronaldo Cutipa-Puma, Cristian Giovanni Coaguila-Quispe, Pablo Raul Yanyachi

**Affiliations:** a School of Electronics Engineering, Universidad Nacional de San Agustín, Arequipa, Peru; b Instituto de Investigación Astronómico y Aeroespacial Pedro Paulet IAAPP-UNSA, Universidad Nacional de San Agustín, Arequipa, Peru

**Keywords:** Prosthesis, EEG, Robotics, Machine Learning, 3D printing

## Abstract

One of the biggest problems faced by amputees is obtaining a suitable low-cost prosthesis. To address this problem, the design and implementation of a transradial prosthesis controlled by electroencephalographic (EEG) signals was carried out. This prosthesis is an alternative to prostheses using electromyographic (EMG) signals, which are very complex and exhausting for the patient to execute.

We collected EEG signal data using the Emotiv Insight Headset, which were then processed to control the movement of the prosthesis, known as the Zero Arm. Additionally, we incorporated Machine Learning algorithms to classify different types of objects and shapes. The prosthesis also features a haptic feedback system, which simulates the function of mechanoreceptors in the skin, providing the user with a sense of touch when using the prosthesis.

Our research has yielded a viable and cost-effective prosthetic limb. We utilized 3D printing and easily obtainable servomotors and controllers, making the prosthesis affordable and accessible.

Performance tests of the Zero Arm prosthesis have yielded promising results. The prosthesis demonstrated an average success rate of 86.67% across various tasks, indicating its reliability and effectiveness. Additionally, the prosthesis has an average recognition rate of 70% for different types of objects, a noteworthy accomplishment.

## Graphical abstract

**Figure:**
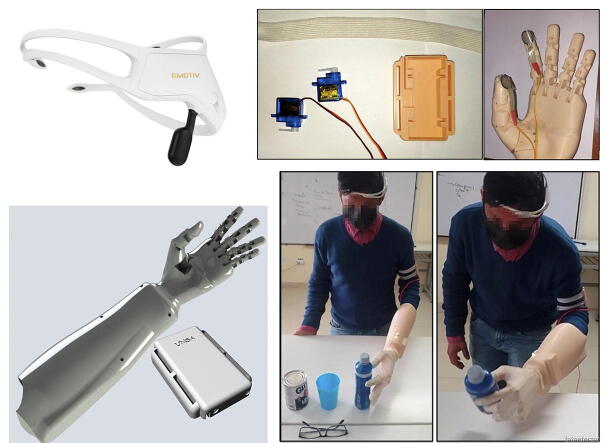

**Specifications table**

**Table:** 

**Hardware name**	Zero Arm
**Subject area**	• Engineering and material science • Medical • Neuroscience • Biological sciences
**Hardware type**	• Biomedical engineering and robotic science
**Closest commercial analog**	Hero Arm
**Open source license**	CC BY 4.0
**Cost of hardware**	USD $600
**Source file repository**	https://doi.org/10.17605/OSF.IO/63S57

## 1 Hardware in context

In the United States, the cost of a prosthetic arm is in the range of USD $5000 for the simplest prosthetic arm and approximately USD $100000 for a neuro-prosthetic model [1]. These costs make good quality prostheses inaccessible to most people, especially in developing countries.

An alternative to the problem of large manufacturing costs in materials for a prosthetic arm is the use of 3D printing technology [2], [3], a technology that comes hand in hand with other benefits such as a reduction in the weight of the prostheses and greater customization of the parts needed [4]. However, low-cost hand prostheses currently have limitations in the amount of movements they can perform [5], [6], [7], unlike high-cost commercial prostheses. These limitations can affect people’s ability to perform everyday tasks and can be an obstacle to a full life.

In recent years, there has been a breakthrough in biosignal classifiers with high accuracies. However, the application of these classifiers in robotic prostheses is still under development. Currently, prostheses are designed depending on the biosignal processed by the classifier [8]. In this sense, our design is able to work with the output signal of any classifier regardless of the type of biosignal.

The use of electromyographic (EMG) signals has been quite common, and almost a standard for the design of transhumeral and transradial prostheses [9], [10], [11], [12]. We present three prominent projects in the field of EMG-controlled robotic hand prostheses. First, Open Bionics offers the robotic hand ”Ada”, a pioneering open-source design that can be 3D-printed and assembled with low-cost electronic components, resulting in a lightweight, durable, and functional prosthesis with a customizable aesthetic design. Second, InMoov develops a complete robotic arm, including hand and forearm, using electronic components and 3D printing to create an accessible and functional prosthesis, with a modular design that allows for adaptations and the incorporation of new technologies. Lastly, the e-NABLE community designs and shares open-source hand prosthesis models for children and adults with amputations or congenital malformations, offering customizable and low-cost solutions through the use of 3D printers and accessible materials. These projects represent significant advances in democratizing access to high-quality and functional robotic prostheses and have the potential to improve the quality of life for countless people around the world. However, in recent years, using electroencephalographic (EEG) signals has been shown to have an equal or better outcome than using EMG signals, especially in patients with neuromuscular disorders [13]. Although the design of prostheses with EEG signals such as those presented in [14], [15], [16] is becoming an alternative in the field, very few of these works are publicly available as open source and fully documented models.

In addition, haptic feedback is a functionality that many arm prostheses do not take into account when they are designed and produced. This functionality is lost when a limb amputation occurs, forcing the user to maintain constant eye contact with the prosthesis when using it [17]. Sensory feedback is essential for grasping objects, manipulation of the human hand, and for efficient motor planning and execution [18].

The use of machine learning algorithms is a promising topic of study in bioengineering and Brain Machine Interfaces (BMI) fields. Recent work has explored the use of machine learning for EEG and EMG signal processing and classification [19], [20], [21]. Machine learning algorithms are increasingly finding application in transhumeral and transradial prostheses. In [22], it is described how machine learning algorithms are used to classify EEG signals for the control of an arm prosthesis, and in [23], EMG signals, classified by machine learning algorithms, are used to generate command controls for arm movements. In this paper, an innovative approach is proposed to enhance the functionality of robotic arm prostheses. The use of machine learning algorithms is suggested to classify signals obtained from force sensors located in the fingers of the prosthesis, aiming to detect shapes and objects. Additionally, EEG signals are analyzed using EmotivXavier software, which provides an intuitive interface for signal processing. This comprehensive approach combines the classification of force signals with the analysis of brain signals to improve interaction and control of the prosthesis, offering a more intuitive and functional experience for users.

We present the construction and operation of Zero Arm, which complies with the features of modern robotic hands and has a low-cost design, under USD $600. This prosthesis uses electroencephalographic (EEG) signals as an alternative to the use of electromyographic (EMG) signals and is a fully replicable open source model. In addition, Zero Arm includes a haptic feedback system based on skin stretching to enhance the user experience.

Finally, as an additional feature, it uses machine learning algorithms and by means of force sensors, it can classify objects into spheres, cubes or cylinders.

## 2 Hardware description

**Fig. 1:**
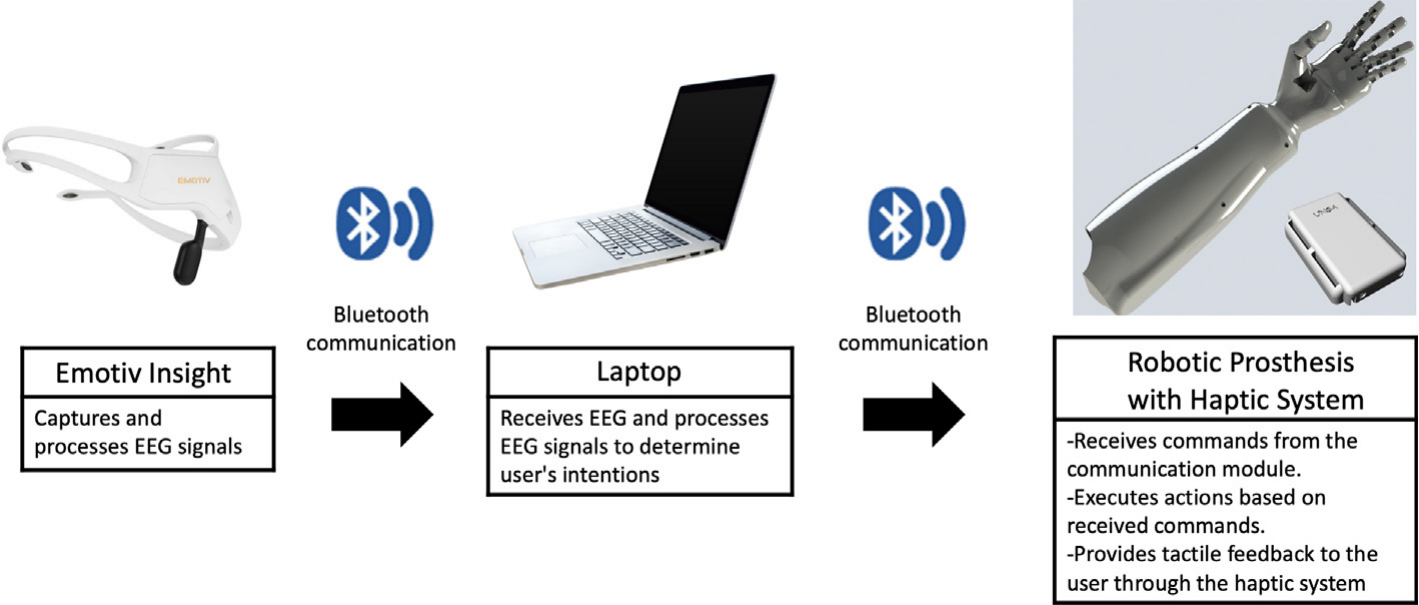
Diagram of the system used for prosthesis movement.

The following section describes the transradial prosthesis implemented in the Zero Arm project. This prosthesis includes four degrees of freedom (DOFs) and a haptic feedback system based on skin stretching. The Emotiv Insight device is used as the EEG signal acquisition system. This device has five channels capable of recording brain wave frequencies. A division of the section into three parts follows. The first part describes the implemented transradial prosthesis. The second part covers the haptic system. Finally, a description of the software used throughout the design is included. Fig. 1 shows the system and devices comprising the prosthesis.

### 2.1 Transradial arm prosthesis

**Fig. 2:**
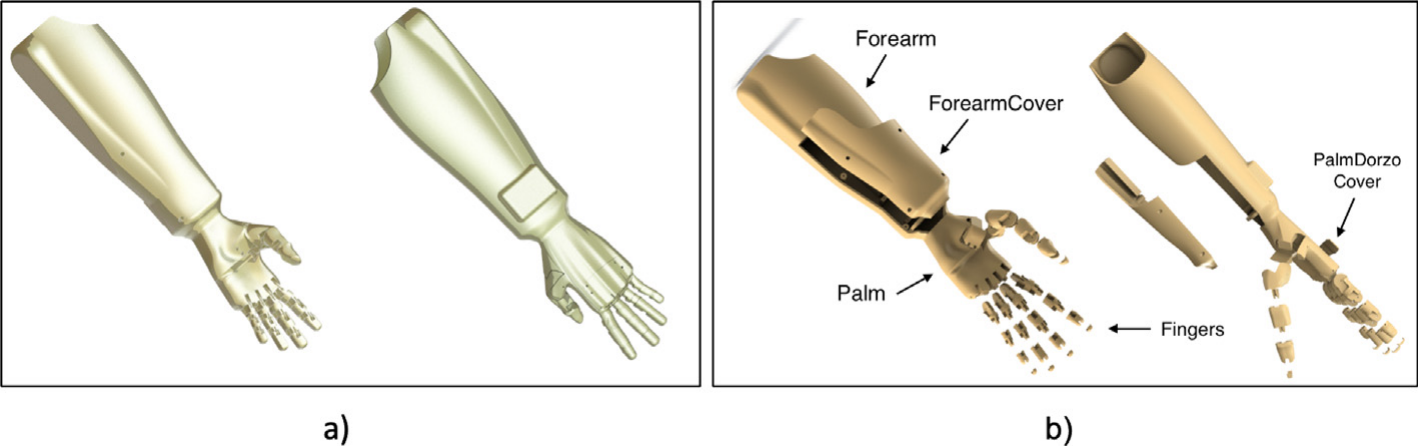
(a) 3D design of the prosthesis. (b) Expanded design of the prosthesis.

The prosthetic arm has been designed in a modular way, with parts made by 3D printing and using PLA filament. The control of the arm has been carried out by means of the Raspberry Pi Pico card. The mechanical design includes 29 modeled parts, including: finger phalanges, palm, palm cap, garter cap, forearm, forearm cover and finger thread guides. All parts are shown in Fig. 2.

The phalanges of the fingers be joined by means of shafts at the joints and retracted by means of tensioned rubber bands. Guide spools and wires have been used to connect the fingers to the servomotors that control them. In total, the arm includes 3 servomotors: one for the joint of the little finger, ring finger and middle finger; one for the index finger and one for the thumb. In addition, a micro servomotor has been included for the opposing movement of the thumb. The estimated weight of the prosthesis as a whole is 1 kg.

**Fig. 3:**
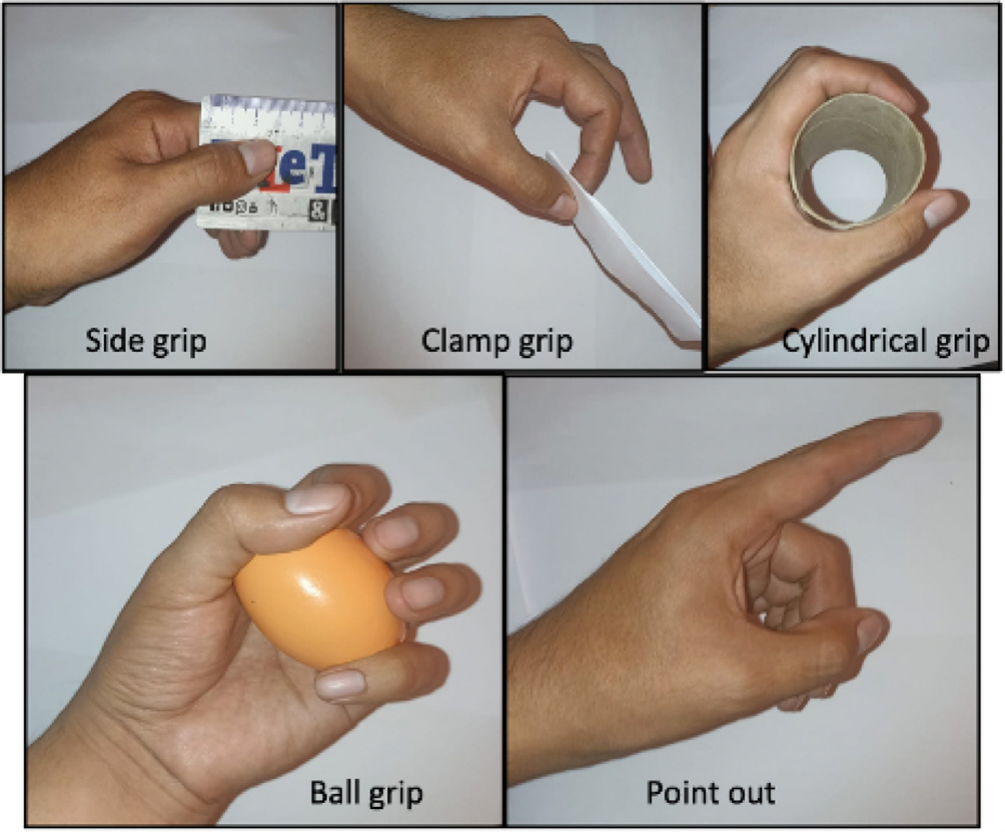
Types of grips that the prosthesis can perform.

The developed prosthesis is capable to perform five types of grips according to the user’s needs, which can be selected by a push button. These grips include side grip, pincer, cylindrical, spherical, and a pointing position, as shown in Fig. 3.

### 2.2 **Haptic system**

**Fig. 4:**
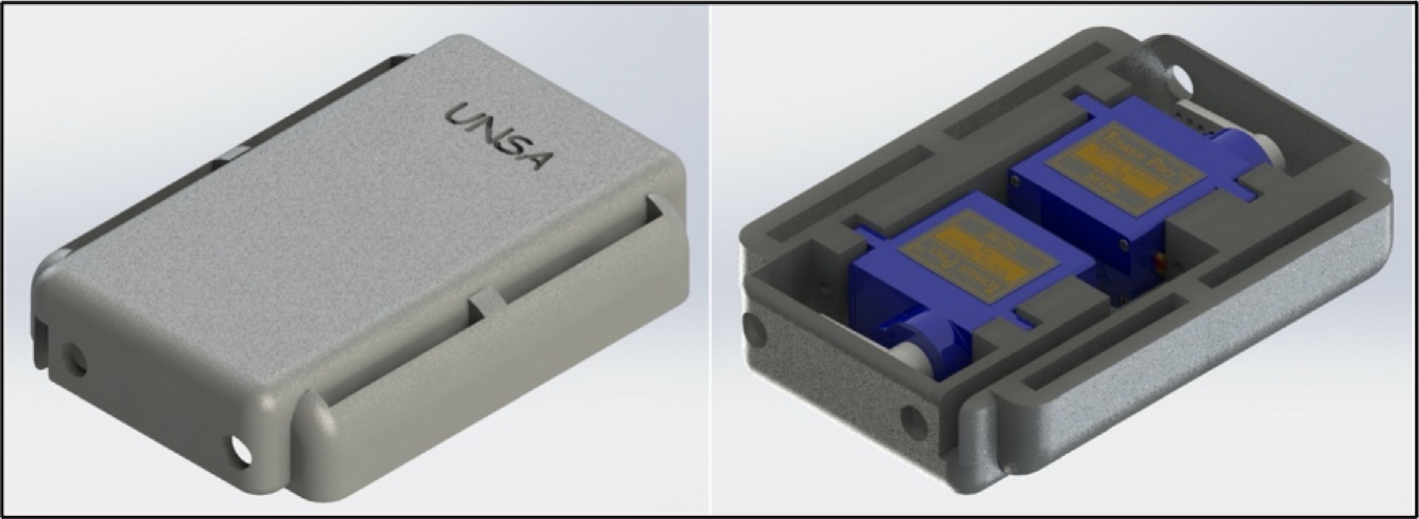
Mechanical structure of the haptic system.

The haptic system consists of a mechanical structure and two servomotors that exert pressure on the user’s skin. The mechanical part of the haptic system is shown in Fig. 4, it is a 3D printed physical component that provides support, stability and protection to the system. It acts as a casing or structure that houses two microservomotors, ensuring their position and allowing their correct operation. In addition, it protects internal components and helps distribute forces and vibrations during system operation.

The values from the force sensors located on the fingers of the prosthesis are read by an Arduino Nano and processed to be mapped to a Pulse Width Modulation(PWM) value that is sent to the servomotors. The intensity registered by the force sensors causes the servomotors to vary their angle of rotation proportionally, which allows the user to feel sensory feedback on the skin when touching an object, simulating the sensation of touching an object.

### 2.3 **Software description**

The system software is composed of three parts: control of the prosthesis grips, proportional control of the haptic system and machine learning for shape detection.

EEG signal classification and the connection between the Emotiv Insight device and the computer are performed using the EmotivXavier software and data is sent to the Raspberry Pi Pico of the prosthesis via a Bluetooth module using the Thonny-Python IDE. The decoded data is used to execute the desired grip. The haptic system is controlled by an Arduino Nano board and a proportional control is executed using the Arduino IDE, where the input values come from the force sensors located on the fingers of the prosthesis and the output values are the rotation angles in their PWM equivalent sent to the servomotors.

Shape detection is performed using a neural network trained with Python to identify the desired objects and everything is exported to the Arduino Nano board once the neural network has been trained and validated.

## 3 Design files summary

**Table:** 

**Design filename**	**File type**	**Open source license**	**Location of the file**	
IndexFinger	3D Model (*.stl)	CC BY 4.0	https://osf.io/9w3fz	
MiddleFinger	3D Model (*.stl)	CC BY 4.0	https://osf.io/uzwtf	
RingFinger	3D Model (*.stl)	CC BY 4.0	https://osf.io/5bjwx	
LittleFinger	3D Model (*.stl)	CC BY 4.0	https://osf.io/s8v7f	
Thumb	3D Model (*.stl)	CC BY 4.0	https://osf.io/gwa8y	
Palm	3D Model (*.stl)	CC BY 4.0	https://osf.io/e6x73	
PalmCover	3D Model (*.stl)	CC BY 4.0	https://osf.io/2af3p	
PalmDorzoCover	3D Model (*.stl)	CC BY 4.0	https://osf.io/exurt	
Forearm	3D Model (*.stl)	CC BY 4.0	https://osf.io/j6mqk	
ForearmCover	3D Model (*.stl)	CC BY 4.0	https://osf.io/ev62k	
ServoGuide	3D Model (*.stl)	CC BY 4.0	https://osf.io/nka7z	
ComModuleCase	3D Model (*.stl)	CC BY 4.0	https://osf.io/gxmvb	
ComModuleCover	3D Model (*.stl)	CC BY 4.0	https://osf.io/ymk58	
ControllerBackPCB	PDF (*.pdf)	CC BY 4.0	https://osf.io/ek4mt	
ControllerFrontPCB	PDF (*.pdf)	CC BY 4.0	https://osf.io/ek4mt	
ComModulePCB	PDF (*.pdf)	CC BY 4.0	https://osf.io/kvxm3	
ComModuleCode	Python (*.py)	CC BY 4.0	https://osf.io/9v6g3	
MainProsthesis	Python (*.py)	CC BY 4.0	https://osf.io/tnj82	
CollectData	Python (*.py)	CC BY 4.0	https://osf.io/r289c	
DataSet	Python (*.py)	CC BY 4.0	https://osf.io/vfmkq	
TrainingClassification	Python (*.py)	CC BY 4.0	https://osf.io/z2xhk	
PyArduinoPlot	Python (*.py)	CC BY 4.0	https://osf.io/jq4ut	
ReadDataset	Python (*.py)	CC BY 4.0	https://osf.io/uswpt	
ArduinoKeras	Python (*.py)	CC BY 4.0	https://osf.io/n5e82	
ReadData	Arduino (*.ino)	CC BY 4.0	https://osf.io/vynw5	
ImplementationML	Arduino (*.ino)	CC BY 4.0	https://osf.io/2mfqv	
SetBluetooth	Arduino (*.ino)	CC BY 4.0	https://osf.io/bjf8c

• IndexFinger: 3D design of three phalanges that make up the index finger.
• MiddleFinger: 3D design of three phalanges that make up the middle finger.
• RingFinger: 3D design of three phalanges that make up the ring finger.
• LittleFinger: 3D design of three phalanges that make up the little finger.
• Thumb: 3D design of three phalanges that make up the thumb.
• Palm: 3D design of the palm.
• PalmCover: 3D design of the coverage of the thenar eminence of the palm.
• PalmDorzoCover: 3D design of the coverage of the back of the palm.
• Forearm: 3D design of the forearm.
• ForearmCover: 3D design of the forearm coverage.
• ServoGuide: 3D design of the wire guides for the servomotors.
• CommunicationModuleCase: 3D design of the protective case of the communication module.
• CommunicationModuleCover: 3D design of the cover of the protective box of the communication module.
• ControllerBackPCB: Template to print the back of the PCB of the controller unit.
• ControllerFrontPCB: Template to print the front of the PCB of the controller unit.
• CommunicationModulePCB: Template to print the back of the communication module PCB.
• CommunicationModuleCode: MicroPython script to send the commands to the prosthesis.
• MainProsthesis: MicroPython script that controls the movements of the prosthesis.
• CollectData: Python script to save training data.
• DataSet: Python script to sort and separate the data for training.
• TrainingClassification: Python script to train the neural network.
• PyArduinoPlot: Python script to establish communication with the Arduino Nano
• ReadDataset: Python script to sort the data received from the Arduino Nano.
• ArduinoKeras: Python script to define neural network parameters.
• ReadData: Arduino code to read the data from the sensors.
• ImplementationML: Arduino code of the neural network resulting from the training.
• SetBluetooth: Arduino code to configure the Bluetooth modules.

## 4 Bill of materials summary

**Table:** 

**Designator**	**Component**	**Number**	**Cost per unit - currency**	**Total cost - currency**	**Source of materials**	**Material type**	
Microcontroller	Raspberry Pi Pico	2	USD $4.23	USD $8.46	https://cutt.ly/MBRRAFX	Other	
Microcontroller	ATmega328P	1	USD $3.47	USD $3.47	https://cutt.ly/dJwYe6a	Other	
Emotiv Insigth	Emotiv Insigth v2.0	1	USD $499.00	USD $499.00	https://cutt.ly/gBRQoaJ	Other	
Bluetooth	Bluetooth HC05	2	USD $2.21	USD $4.42	https://cutt.ly/uBRniKC	Other	
Switches	Mini Rocker Switch 2 PIN ON–OFF	1	USD $0.07	USD $0.07	https://cutt.ly/t6fKd86	Other	
Connectors	Pin Female	10	USD $0.699	USD $6.99	https://cutt.ly/PJwOtAd	Other	
Connectors	Pin Male	3	USD $0.499	USD $1.49	https://cutt.ly/LJwO516	Other	
Micro servomotor	MG996R	3	USD $2.34	USD $7.02	https://cutt.ly/nBdDGwd	Other	
Servomotor	XL4015	3	USD $2.75	USD $8.25	https://cutt.ly/qBdFmLT	Other	
Voltage regulator	STM32	1	USD $1.88	USD $1.88	https://cutt.ly/1BdLR5E	Other	
RGB LED	STM32	1	USD $0.95	USD $0.95	https://cutt.ly/QBdGLRG	Other	
Push Button	Modulo Touch Switch	1	USD $0.35	USD $0.35	https://cutt.ly/1BdLSn7	Other	
Jumpers	Dupont Line Cable	40	USD $0.016	USD $0.64	https://cutt.ly/5BdZMm9	Other	
USB cable	Micro-USB cable	2	USD $1.58	USD $3.16	https://cutt.ly/ABd1ta9	Other	
Suspenders	Elastic band 2 mm	1	USD $0.68	USD $0.68	https://cutt.ly/ABvDlpT	Rubber	
Screws	M2-10 mm	1	USD $1.73	USD $1.73	https://cutt.ly/fBRPdRa	Metal	
PLA	1 kg filament	1	USD $10.39	USD $10.39	https://cutt.ly/bBRIJsx	Other	
Rod	Steel rod	1	USD $1.25	USD $1.25	https://cutt.ly/CBRvMHn	Metal	
Surgical glove	Glove	1	USD $2.96	USD $2.96	https://cutt.ly/hBRUp1I	Rubber	
Cables	UL2464 24AWG	1	USD $2.52	USD $2.52	https://cutt.ly/eBvGPSS	Other	
Battery	Lipo battery 144001 2s	2	USD $10.21	USD $20.42	https://cutt.ly/J6fKVZB	Other	
Nylon	Nylon	1	USD $0.97	USD $0.97	https://cutt.ly/CBdL6BQ	Plastic	
Force sensor	FSR402	3	USD $2.71	USD $8.13	https://cutt.ly/vBdKFu0	Other	
Bra garters	Bra garters	2	USD $1.57	USD $3.14	https://cutt.ly/1BdHA1G	Rubber	

## 5 Build instructions

The Zero Arm build process consists of three main steps: (1) 3D print all the STL files, (2) implement the printed circuit boards (PCBs) of the controller and communication module, (3) assemble all the components and connect them to the PCBs.

To implement a prosthesis on the right side, a mirror inversion of all 3D parts of the design must be performed. Regarding the electronics, the connections remain the same for both sides, which means that the codes work for both extremities without the need for additional adjustments. This adaptability of the design allows for a simple and effective solution for those users who require an arm prosthesis on their right side.

To carry out this process, hand tools such as screwdrivers, pliers, drill and soldering iron are required. Safety glasses are also recommended.

### 5.1 3D Printing

**Fig. 5:**
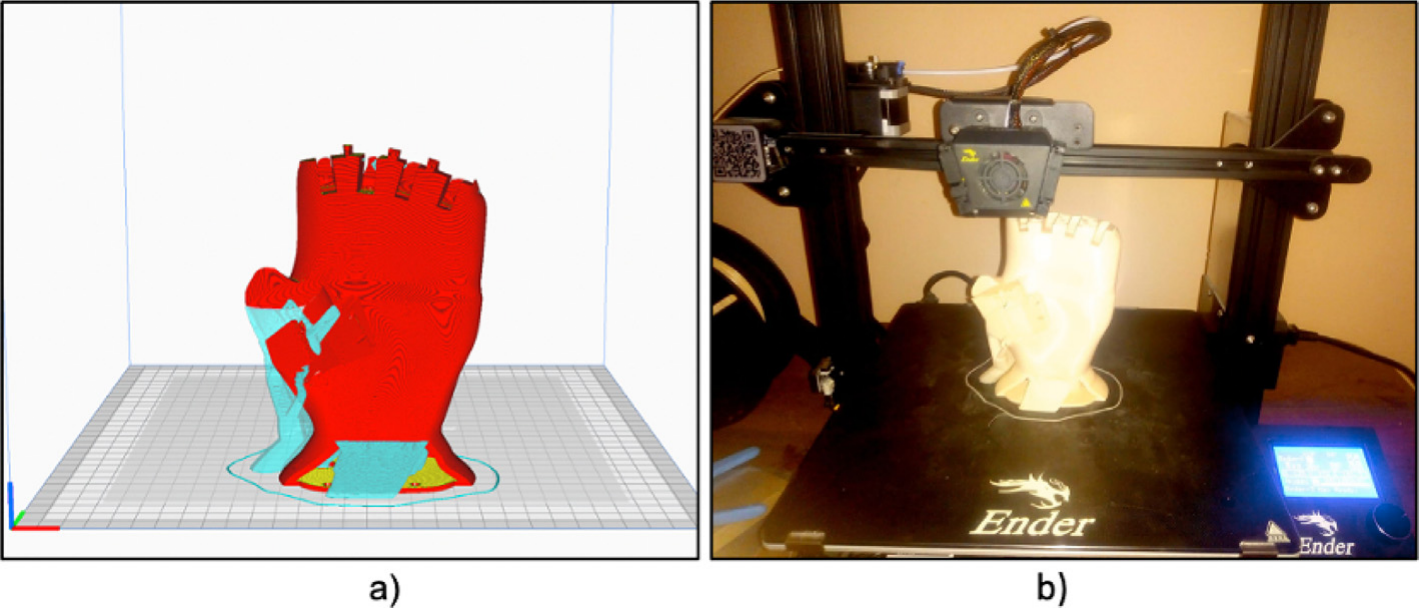
(a) 3D printing preview of the palm of the hand, (b) 3D printing using a commercial FDM printer.

To begin, we download the 3D files in STL format and then generate the G-code needed for printing. We used an Ender 3 Max printer and the filament was PLA, as shown in Fig. 5, due to the ease of printing. However, ABS filament can be used to obtain more durable parts, although this leads to greater difficulty in printing.

To begin the construction process, follow these steps:

1. Prepare the 3D model of the robotic hand prosthesis using a 3D modeling software and import it into the 3D slicing software.
2. Configure the printing parameters as described below:
  • Nozzle Diameter: 0.4 mm
  • Layer Height: 0.2 mm
  • Infill: 20%
  • Supports: Tree-type
  • Printing Temperature: 200–220 °C
  • Printing Speed: 50–60 mm/s
  • Print Bed: Tempered Glass
  • Initial Layer: Skirt
3. Start the 3D printing process by clicking ”Print” in the 3D slicing software. Monitor the progress of the print to ensure it is running smoothly.
4. Once the printing is complete, remove the printed parts from the print bed. Use caution when handling the parts to avoid any damage.
5. Carefully remove the support structures using pliers or a cutter. Take your time to avoid damaging the printed parts during this process.
6. Clean the printed parts to remove any remaining support residues or debris. You can use tools like sandpaper or a brush to smoothen the surfaces if desired.
7. Repeat the printing process for any additional parts required for the assembly of the robotic prosthesis.

### 5.2 Implement the PCBs

**Fig. 6:**
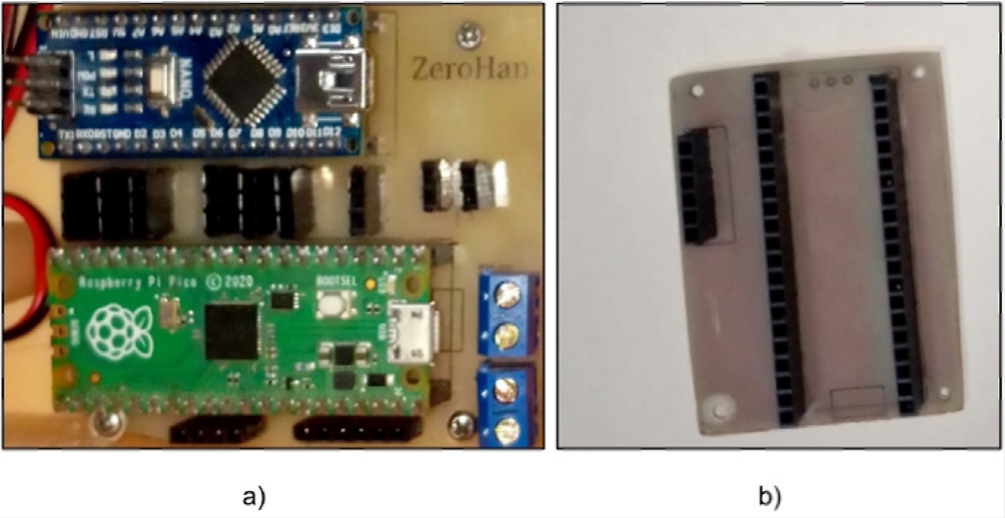
(a) PCB of the prosthesis controller unit, (b) PCB of the communication module.

Laser print the files ControllerBackPCB.pdf, ControllerFrontPCB.pdf and CommunicationModulePCB.pdf proceed with the silkscreen printing method for both PCBs, make the holes where appropriate and solder the components in the right place as shown in Fig. 6, you can also choose to send it to a specialized company like EASYEDA, the details and quality will be much better but the price will be higher.

### 5.3 Assembly of 3D printed parts

**Fig. 7:**
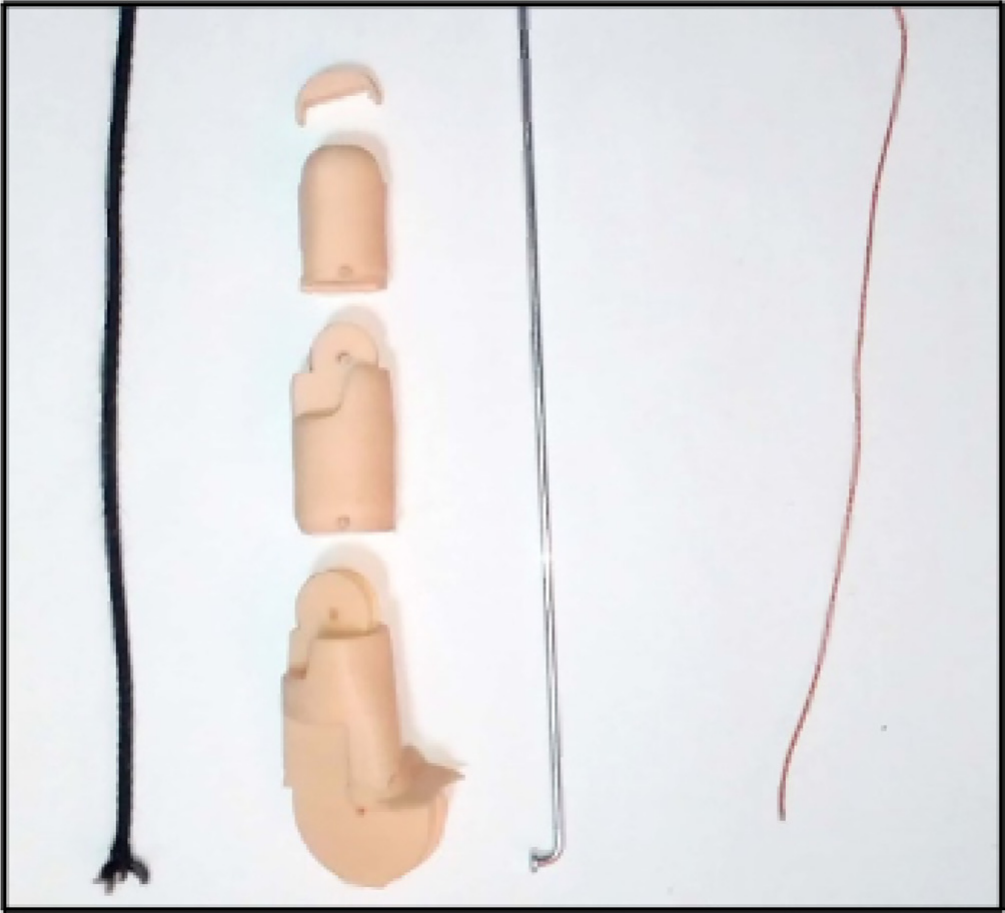
Materials to implement the fingers of the prosthesis.

**Fig. 8:**
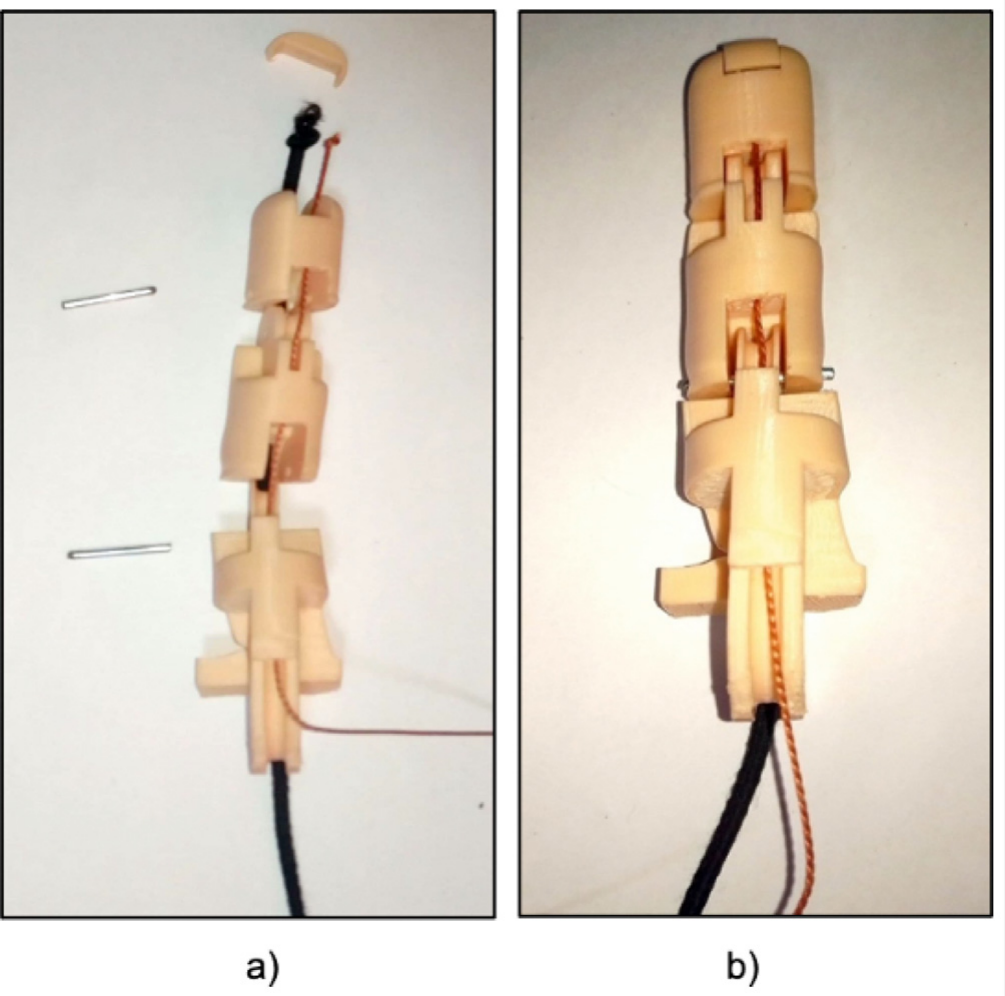
(a) Location of the thread and elastic, (b) Assembly of a finger of the prosthesis.

Each finger consists of 3 phalanges and the procedure is the same for the 5 fingers for this we need thick thread or nylon, elastic band and a steel rod as shown in Fig. 7. To correctly implement each finger we must follow these steps. First, arrange the phalanges and insert the rubber band and the nylon through the holes, secure them at the tip of the finger, now we must join the phalanges with the steel rods, they serve as an axis, as shown in Fig. 8.

**Fig. 9:**
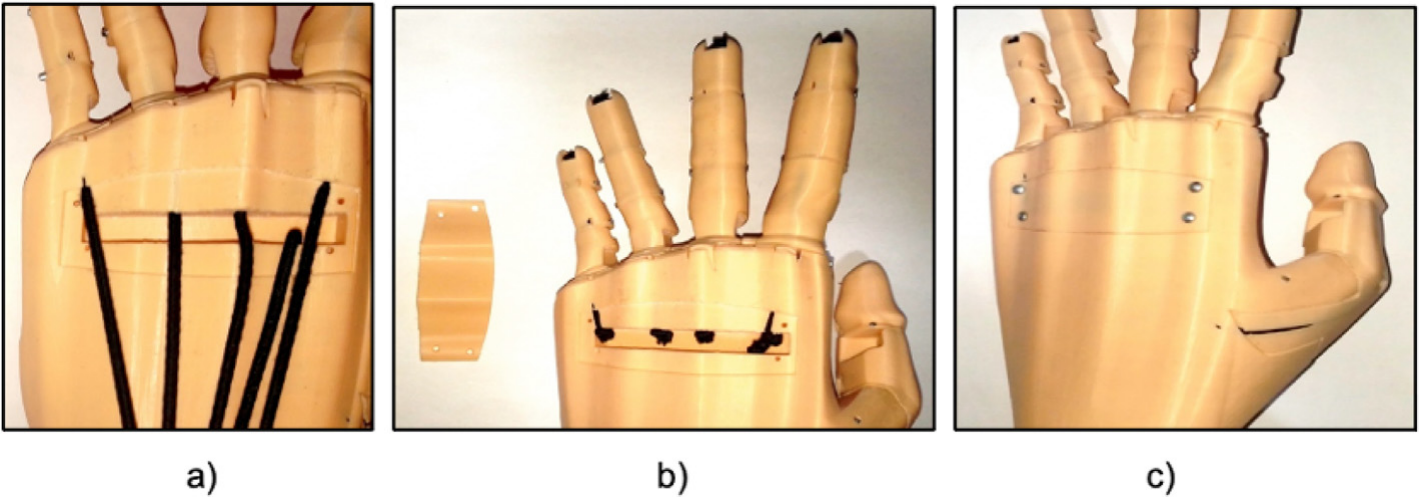
Finger and palm joint of the prosthesis.

After repeating the procedure for the five fingers, we must attach the fingers to the palm, tighten the bands and cover the back of the palm, as shown in Fig. 9.

**Fig. 10:**
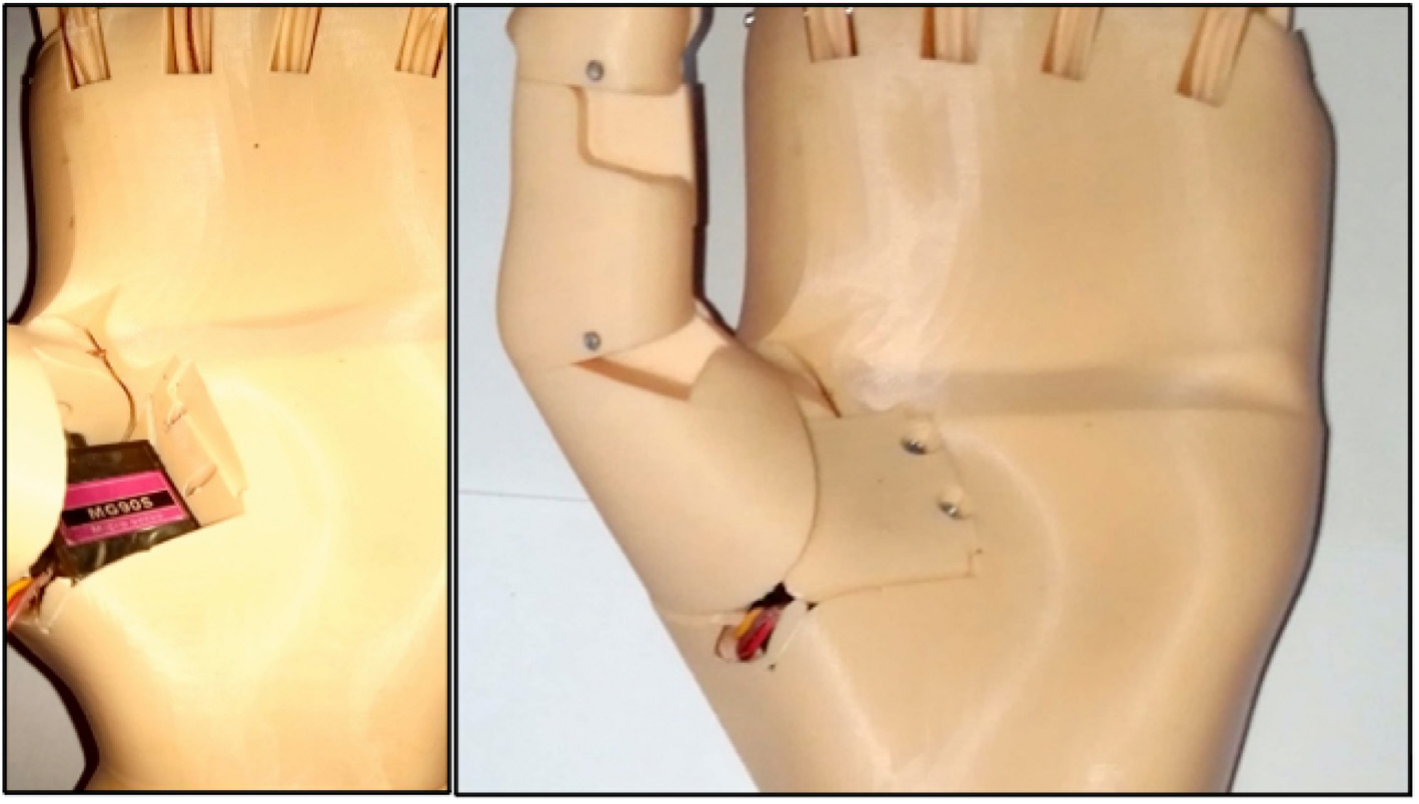
Location of the micro servomotor in the palm of the prosthesis.

Then, place the micro servomotor on the palm and cover it to keep it fixed as shown in Fig. 10, we must be careful with the angle of rotation of the micro servomotor.

**Fig. 11:**
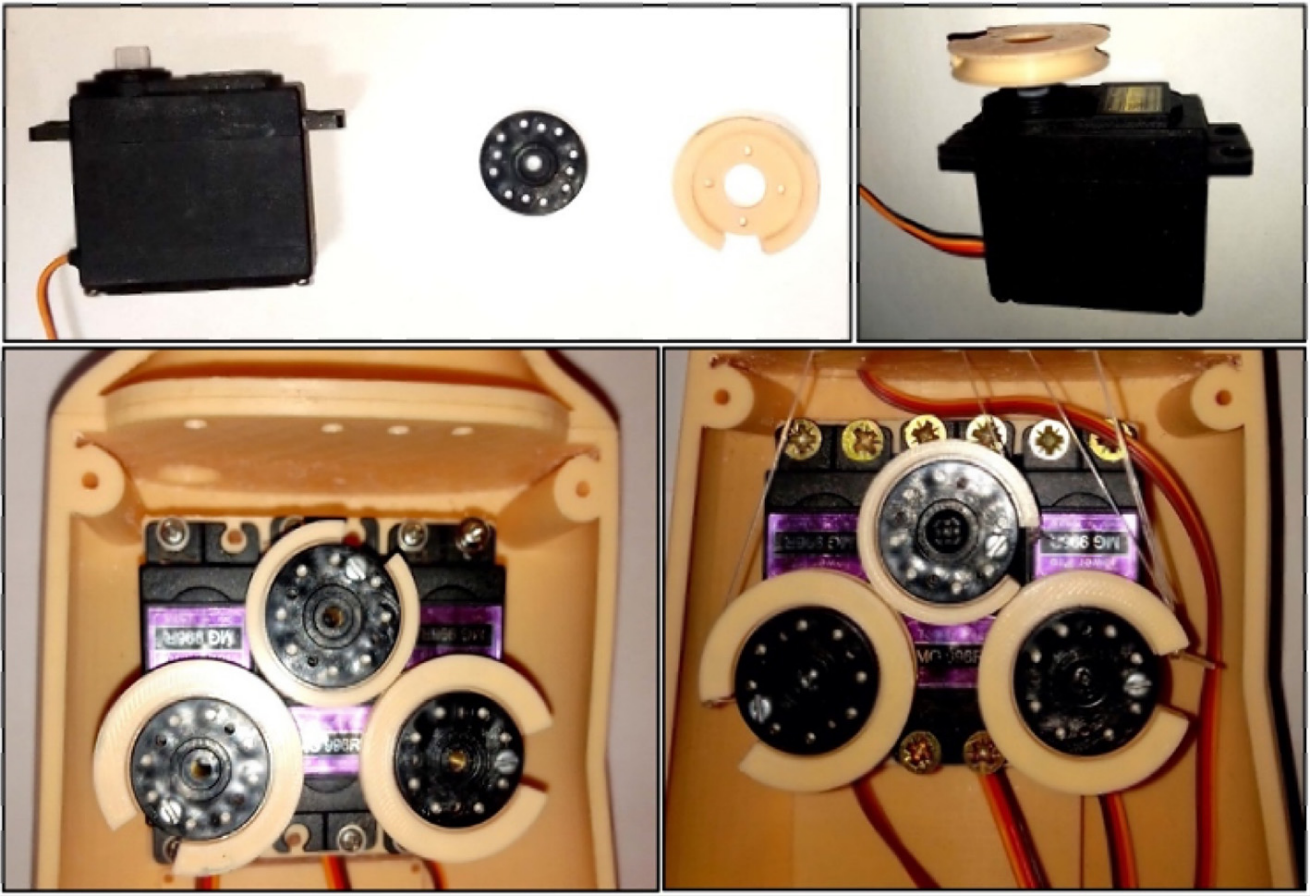
Implementation of the servomotors in the forearm of the prosthesis.

The next step in the assembly process of our designed arm prosthesis involves placing the 3D printed guides onto the servomotors and attaching them to the inside of the forearm. Then, each finger’s nylon is fitted to its respective guide, as illustrated in Fig. 11.

**Fig. 12:**
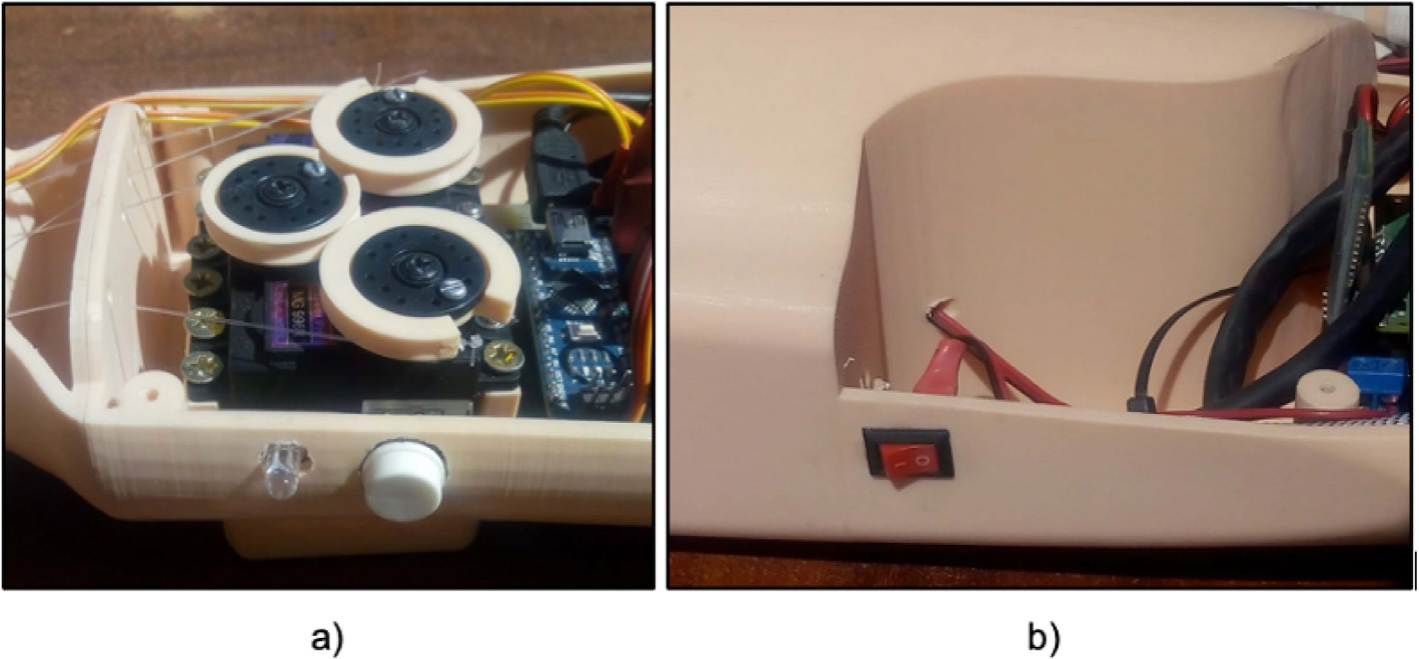
(a) Location of the push button and RGB LED, (b) Location of the main switch of the prosthesis.

Subsequently, position the RGB LED, push button, and switch in their respective designated locations, as depicted in Fig. 12.

**Fig. 13:**
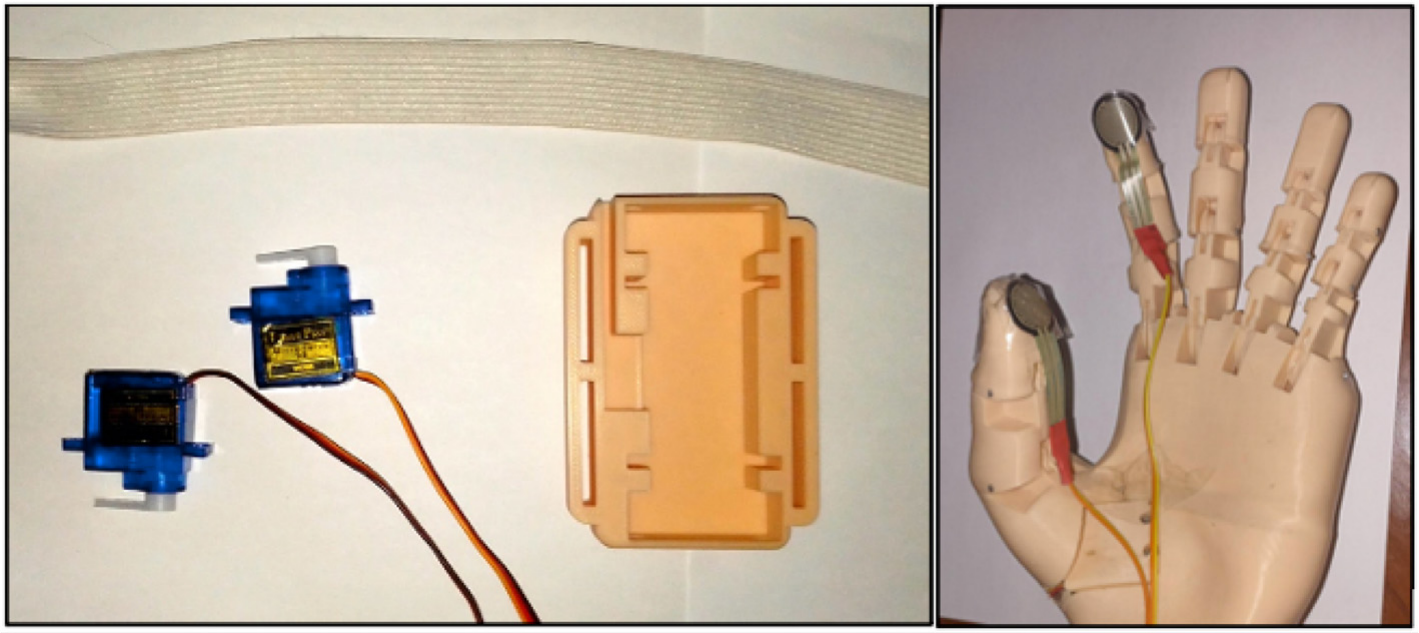
Implementation of the haptic system.

Next, we implement the haptic system by placing the micro servomotors inside the 3D printed component and securing them with screws. Then, the force sensors are glued onto the index fingers, as shown in Fig. 13.

**Fig. 14:**
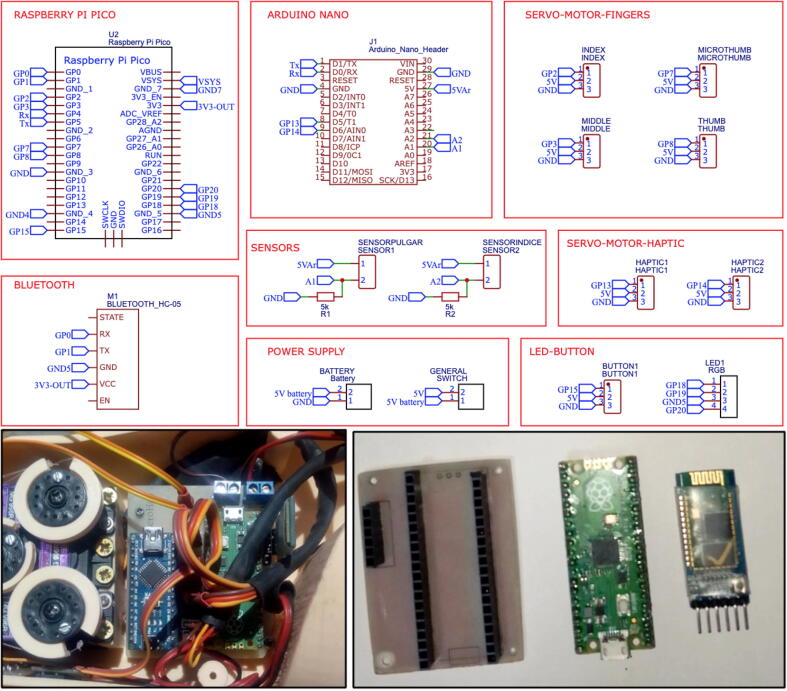
Connection diagram of the components to the controller card of the prosthesis.

Finally, we proceed to connect the servomotors, RGB LED, push button, battery, and sensors to the PCB of the controller unit. Additionally, we need to connect a Raspberry Pi Pico and a Bluetooth module to the PCB of the communication module, as depicted in Fig. 14.

## 6 Operation instructions

Consider the following steps for the correct use or manipulation of the prosthesis.

**Fig. 15:**
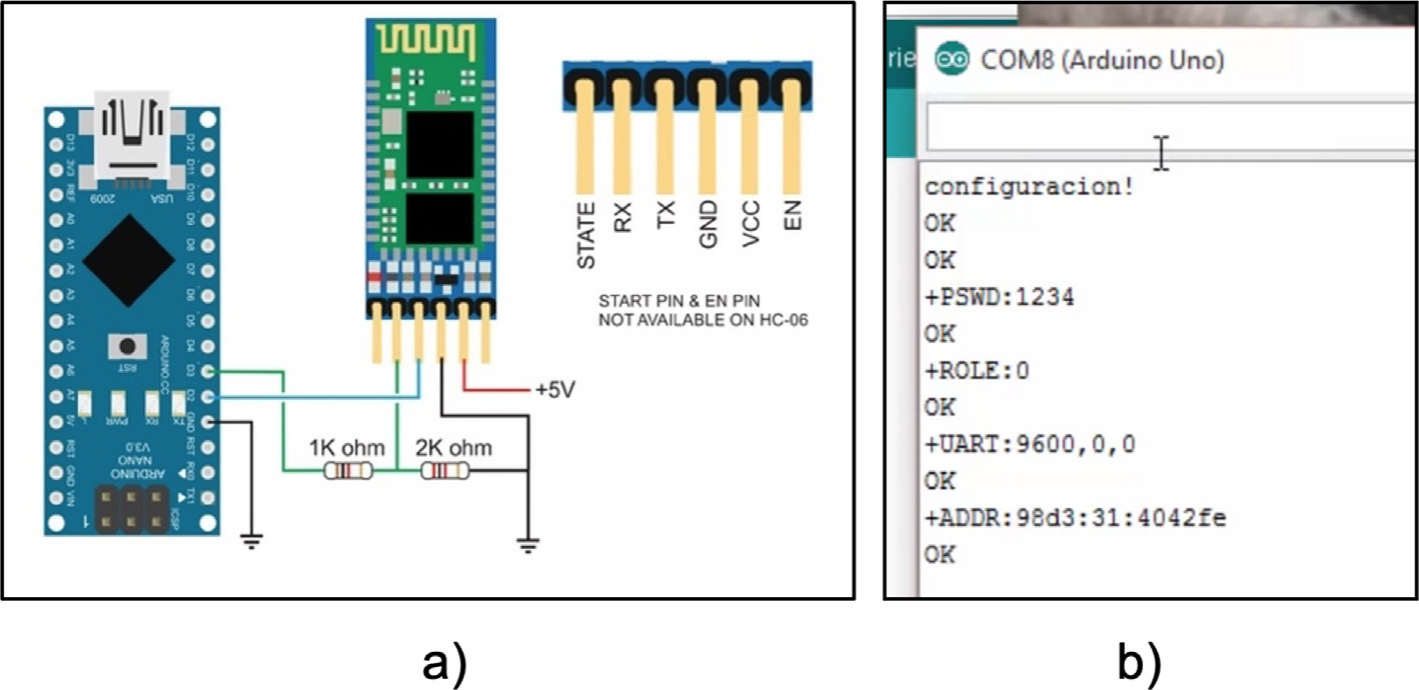
(a) Pairing of Bluetooth modules, (b) AT commands to obtain the address.

Step 01: We pair the Bluetooth modules, this step is only done the first time, then the connection is automatic, for this we need a breadboard, the Arduino Nano and the Bluetooth modules, assemble the circuit shown in Fig. 15(a), then proceed to run the SetBluetooth code, open the serial monitor and send the command AT + ROLE = 0, to configure a module as a slave, additionally we get the module address with the command AT + ADDR? we save the address and do the same for the other Bluetooth module, with the difference that we will send different commands, AT + ROLE = 1 and AT + BIND=<ADDR>, as shown in Fig. 15(b), in this way we set it as master and command it to connect to the Bluetooth slave with the address we saved from the module we configured first. Tutorials for this step can be found on the official Arduino website.

**Fig. 16:**
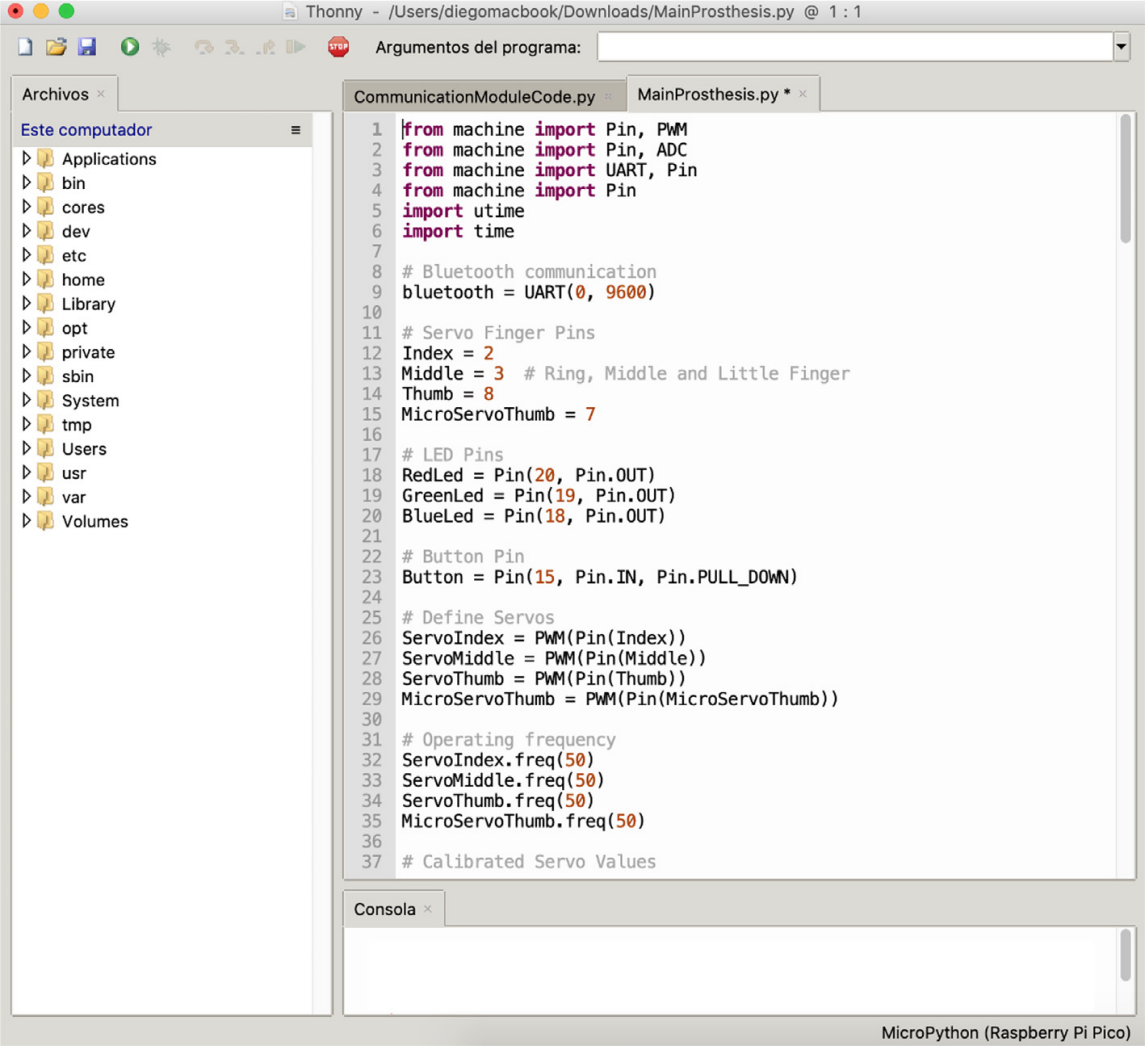
Code loading on Raspberry Pi Pico boards.

Step 02: Load the CommunicationModuleCode and MainProsthesis codes to the Raspberry Pi Pico boards, for this we use the Thonny IDE and save the files inside the Raspberry memory with the name ”main” so that it runs automatically when energizing the prosthesis, as shown in Fig. 16.

**Fig. 17:**
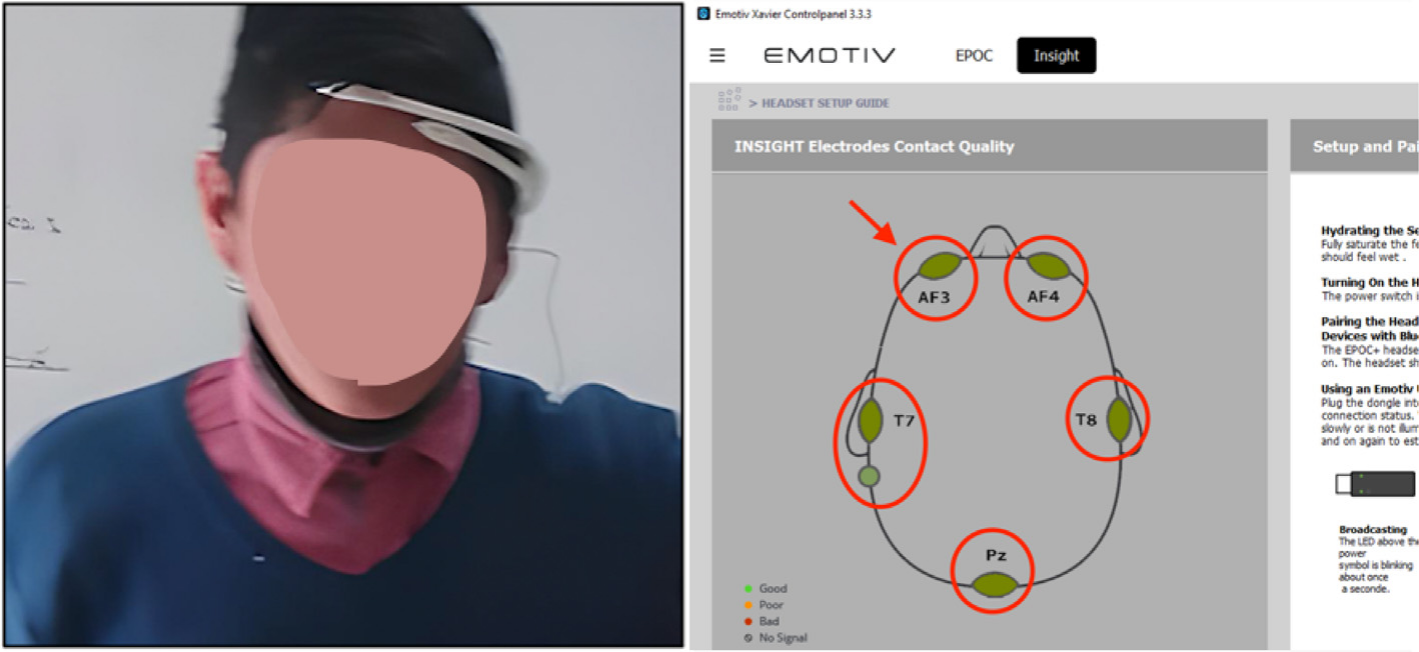
User using the headset correctly and an optimal connection with all the electrodes in green.

Step 03: Turn on and Ensure that the Emotiv Insight is correctly placed on the user’s head. Verify that the electrodes are in the appropriate locations on the scalp, and ensure that the device is powered on and connected to the computer as shown in Fig. 17. Follow the instructions:

1. Open the EmotivXavier software and select the Emotiv Insight as the input EEG device.
2. In the EmotivXavier software, click on the *Stimulus* option in the top toolbar and select *Visual Stimulus* from the dropdown menu.
3. A new window will open where you can choose the type of visual stimulus you want to present. You can select from various types of visual stimuli, such as images, text, or videos.
4. Choose the visual stimulus you want to use for training and adjust the stimulus properties according to your needs, such as duration, presentation frequency, and position on the screen.
5. Next, configure the training properties in the *Training* section within the visual stimulus window. Here, you can set the training duration, the number of stimulus repetitions, and other related parameters.
6. Once you have configured all the options, click the *Start Training* button in the visual stimulus window.
7. During the training session, the visual stimulus will be presented to the user, and the Emotiv Insight will record the user’s brain activity in response to the stimulus.

**Fig. 18:**
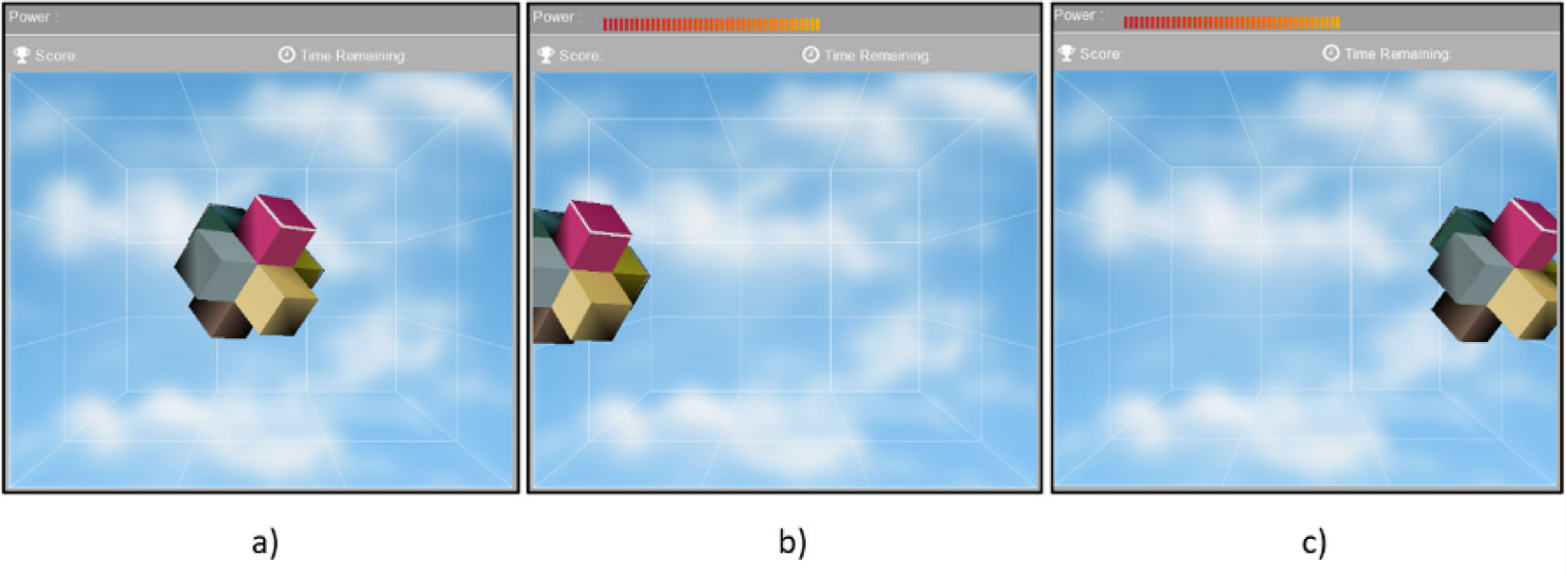
(a) Training in the neutral state, (b) training of the left command, (c) training of the right command.

Step 04: Perform the training of EEG signals, we recommend training two commands which will be left and right, it is also necessary to train the neutral state, as shown in Fig. 18.

Step 05: Encode and transmit the characters to the Prosthesis. In this step, we utilize the Emotiv Xavier Emokey software to encode the commands for the prosthesis. The software provides a user-friendly interface for encoding and transmitting the desired actions.

To encode the commands, we follow the instructions provided by the Emotiv Xavier Emokey software. We select specific actions, such as left or right movements, and the software assigns the corresponding characters ’a’ and ’b’, respectively, to represent these actions.

Once the commands are encoded using Emotiv Xavier Emokey, we proceed to execute the CommunicationModuleCode in the Thonny IDE. This code module establishes the communication link between the software and the prosthesis.

In the Thonny IDE, we have a command window where we enter the encoded characters. For example, to instruct the prosthesis to perform a left movement, we enter the character ’a’. Similarly, we enter the character ’b’ for a right movement. These encoded characters are then transmitted through the communication module from the command window to the prosthesis.

**Fig. 19:**
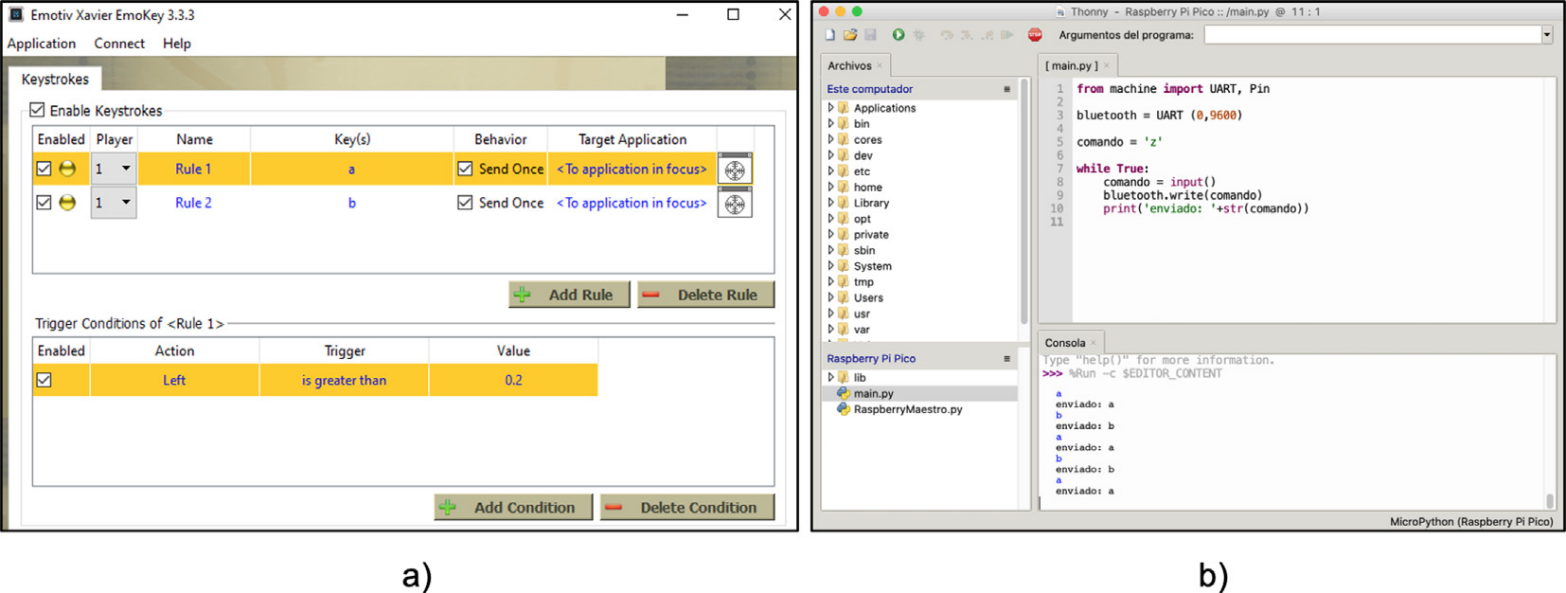
(a) Coding of the left and right commands, (b) Execution of the code in the Thonny IDE.

To provide a visual representation, Fig. 19(a) illustrates the process of encoding the commands using the Emotiv Xavier Emokey software, where the characters ’a’ and ’b’ are assigned to left and right movements, respectively. Fig. 19(b) depicts the transmission of the encoded characters from the Thonny IDE command window to the prosthesis via the communication module.

It is worth noting that the Emotiv Xavier Emokey software simplifies the encoding process, allowing users to intuitively assign characters to specific actions, thereby enhancing the overall usability and control of the prosthesis.

Step 06: Place and fix the prosthesis, we must protect the stump with a bandage, additionally to keep it fixed we must adjust the straps, it is important to ensure comfort to avoid incidents with the prosthesis.

In the apparent haptic system, the mechanical part is placed on the arm at the biceps level. It is important to note that the system does not cause harm to the user, as it uses small servomotors and the pressure they exert on the skin can be varied according to the user’s comfort. Additionally, in case the user prefers not to have direct contact with the skin, the system can be placed on a shirt or sleeve to ensure that it does not cause any harm. This flexibility in the system’s design allows us to adapt to the needs and preferences of each user, ensuring a comfortable and safe user experience.

Step 07: Turn on the prosthesis with the main switch shown in Fig. 12(b), then verify that the prosthesis opens and closes the hand correctly, this starting movement allows us to verify the correct functioning, in case of any anomaly the tensioned nylon should be adjusted or released.

**Fig. 20:**
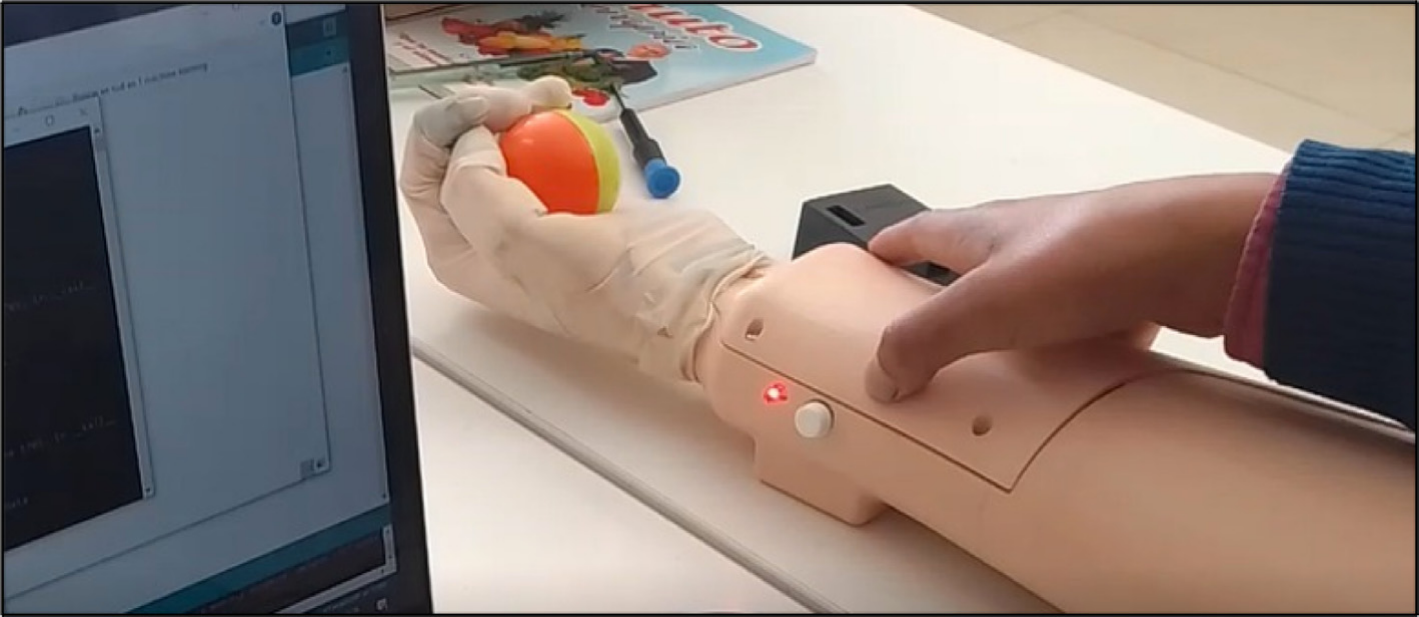
Interaction with the button to change the grip type.

Step 08: Interact with the push button to change the grasping mode of the prosthesis by considering the color of the RGB LED to choose desired grasping as shown in Fig. 20.

• The red color is equivalent to the closed position of the hand.
• The green color is equivalent to the gripper grip.
• The blue color is equivalent to the side clamp grip.
• The yellow color is equivalent to the spherical grip.
• The magenta color is equivalent to the cylindrical grip.
• The white color is equivalent to the signal position.

**Fig. 21:**
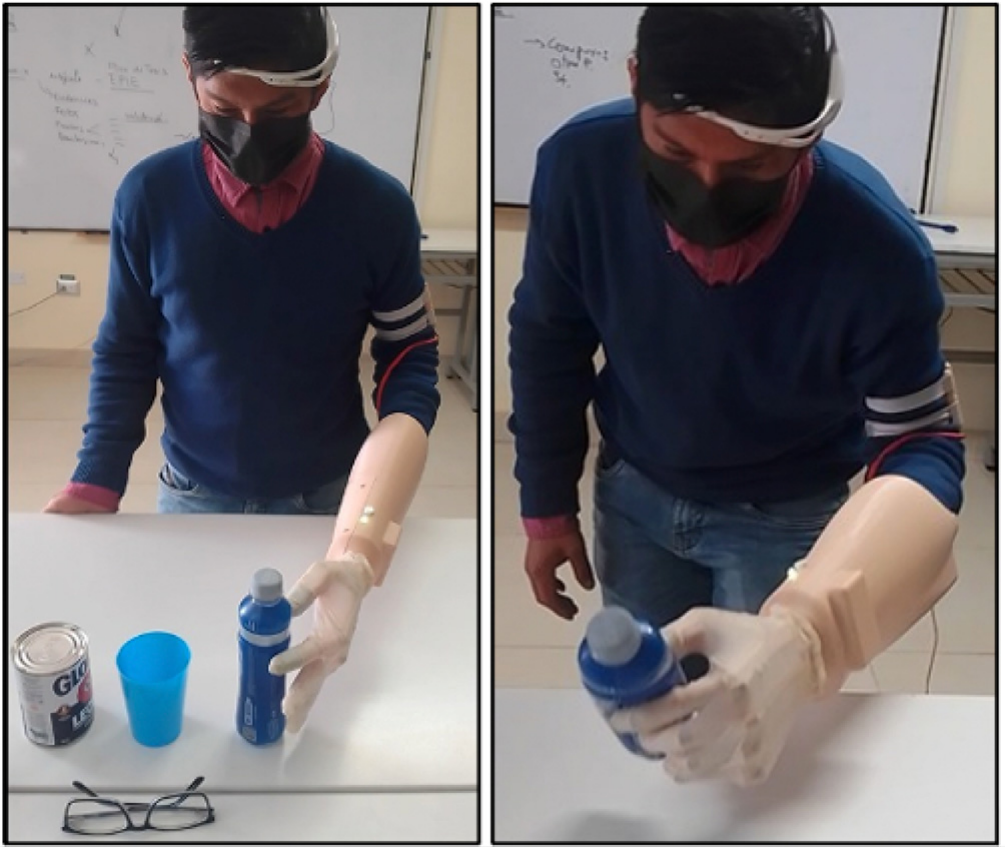
Prosthesis in use.

Step 09: I sent the commands to execute or stop the prosthesis grip at will, as shown in Fig. 19, up to this point we have a useful prosthesis for everyday tasks, however, we still need to configure the machine learning and haptic sense. (See Fig. 21)

**Fig. 22:**
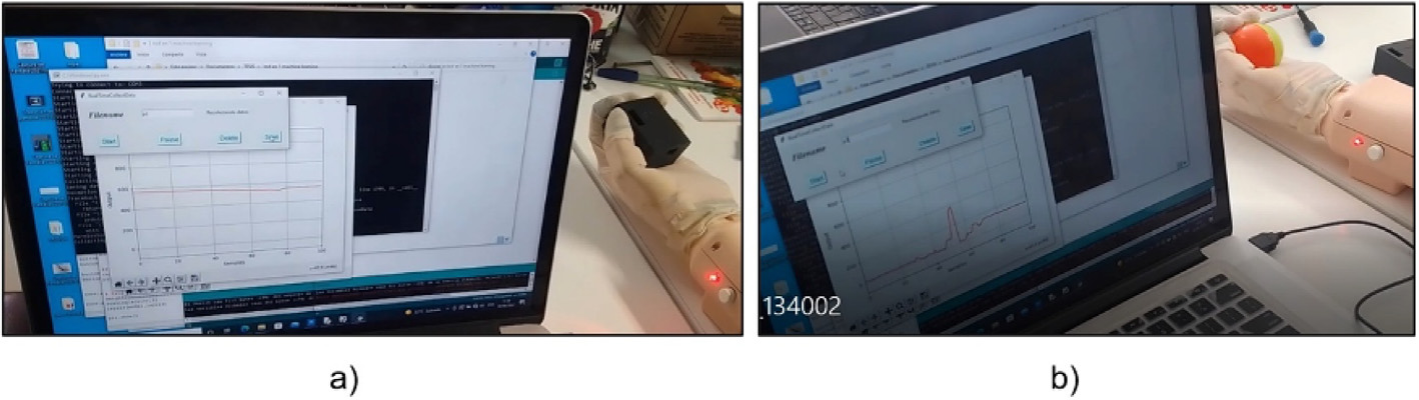
(a) Saving data holding a cube, (b) Saving data holding a sphere.

Step 10: Data collection for machine learning, we must, run the CollectData script in Python, an HMI will be displayed and we save the values when the prosthesis is without objects, then we perform the same procedure holding different objects, as shown in Fig. 22.

For the training and classification process of the robotic arm prosthesis, we recorded 100 continuous measurements for each object. The data collection involved holding the object with the prosthesis while capturing force sensor data. The aim was to gather the necessary information for precise object identification and classification.

The data collection was conducted in collaboration with prosthesis users who performed various actions, such as grasping objects of different shapes and sizes. During these actions, force sensor data was recorded to capture information about force distribution at different contact points.

**Fig. 23:**
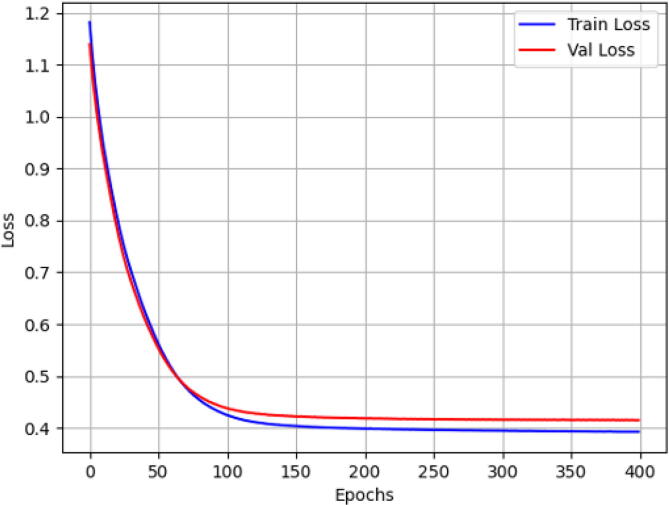
Training and validation of the neural network.

Step 11: Training and validation of the machine learning, run the DataSet and TrainingClassification scripts in the corresponding order, finished the training you should verify that the error in the validation curve tends to zero, as shown in Fig. 23.

In the validation of a machine learning model’s training, a technique known as the validation curve is often used to determine the model’s performance on data that was not used to train it. The validation curve is a graph that shows the relationship between the model’s performance and the number of training data points used to train it.

The error in the validation curve is the difference between the model’s performance on the training data and the model’s performance on the validation data. Generally, the goal is to minimize the error in the validation curve to obtain a model that generalizes well and can predict accurately on new data.

To achieve good model generalization, the error in the validation curve should be close to zero. However, this does not mean that the error has to be exactly zero, as there may be some degree of overfitting or underfitting in the model. Overfitting occurs when the model fits too closely to the training data and does not generalize well, resulting in low error on the training data but high error on the validation data. On the other hand, underfitting occurs when the model is too simple and cannot capture the complexity of the data, resulting in high error on both the training and validation data.

In summary, the goal is to find a balance between overfitting and underfitting, and achieve an error in the validation curve as close to zero as possible to obtain a model that generalizes well and can predict accurately on new data.

**Fig. 24:**
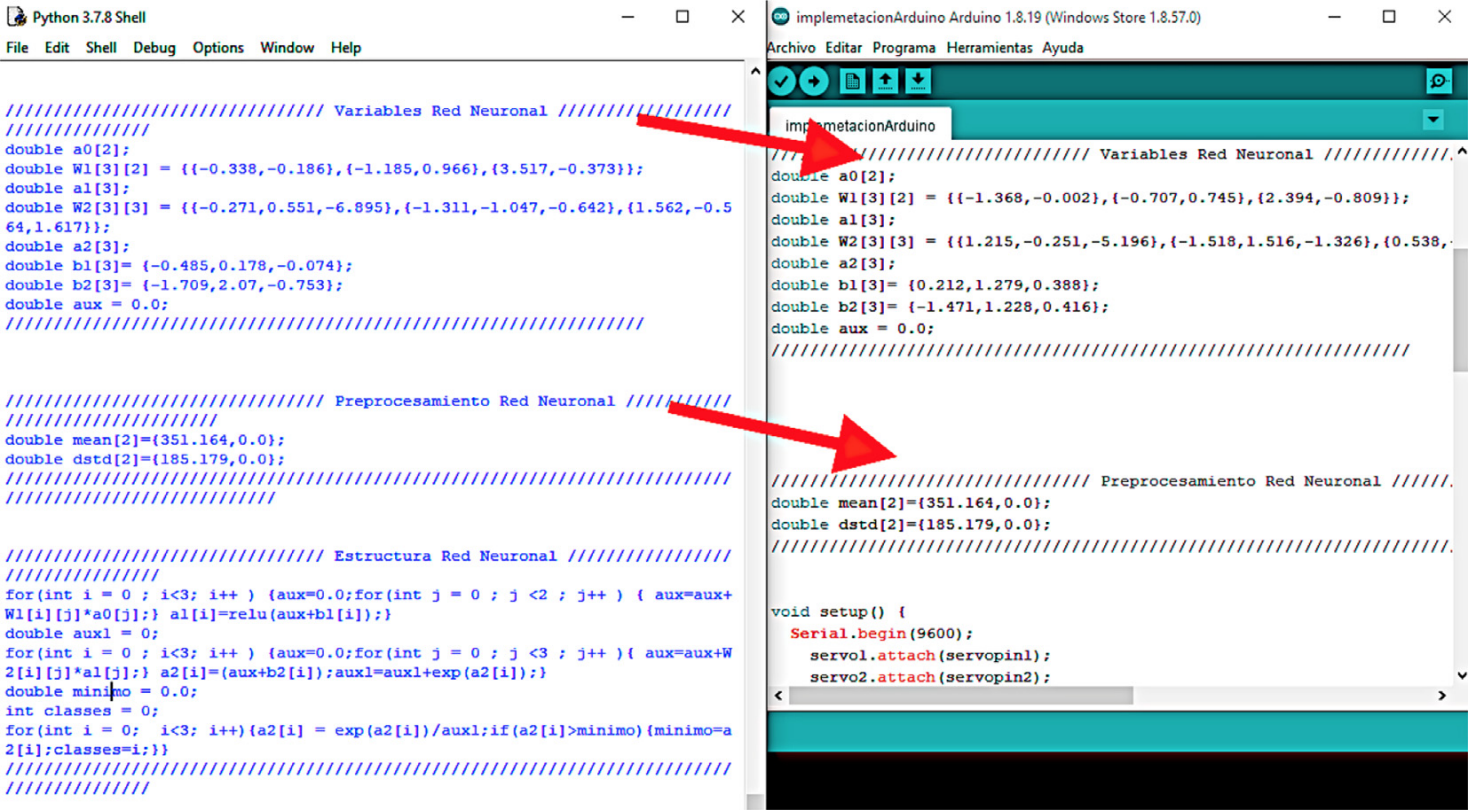
Machine learning implementation on the Arduino Nano.

Step 12: We implement machine learning on the Arduino Nano. Finally, we need to copy the results shown by the command window to the Arduino. We only need to replace the changing values in the ImplementationML code, as shown in Fig. 24. Additionally, further configurations can be made in the network architecture, but this may become confusing and tedious.

## 7 Validation and characterization

To validate the Zero Arm prosthesis, we rely on the international competition Cybathlon, where the best prostheses of the market and new prototypes are gathered. This assures us that our prosthesis can perform the same functions as a high-cost prosthesis. Participants in this test gave written consent to perform these tests.

### 7.1 Prosthesis grips

**Fig. 25:**
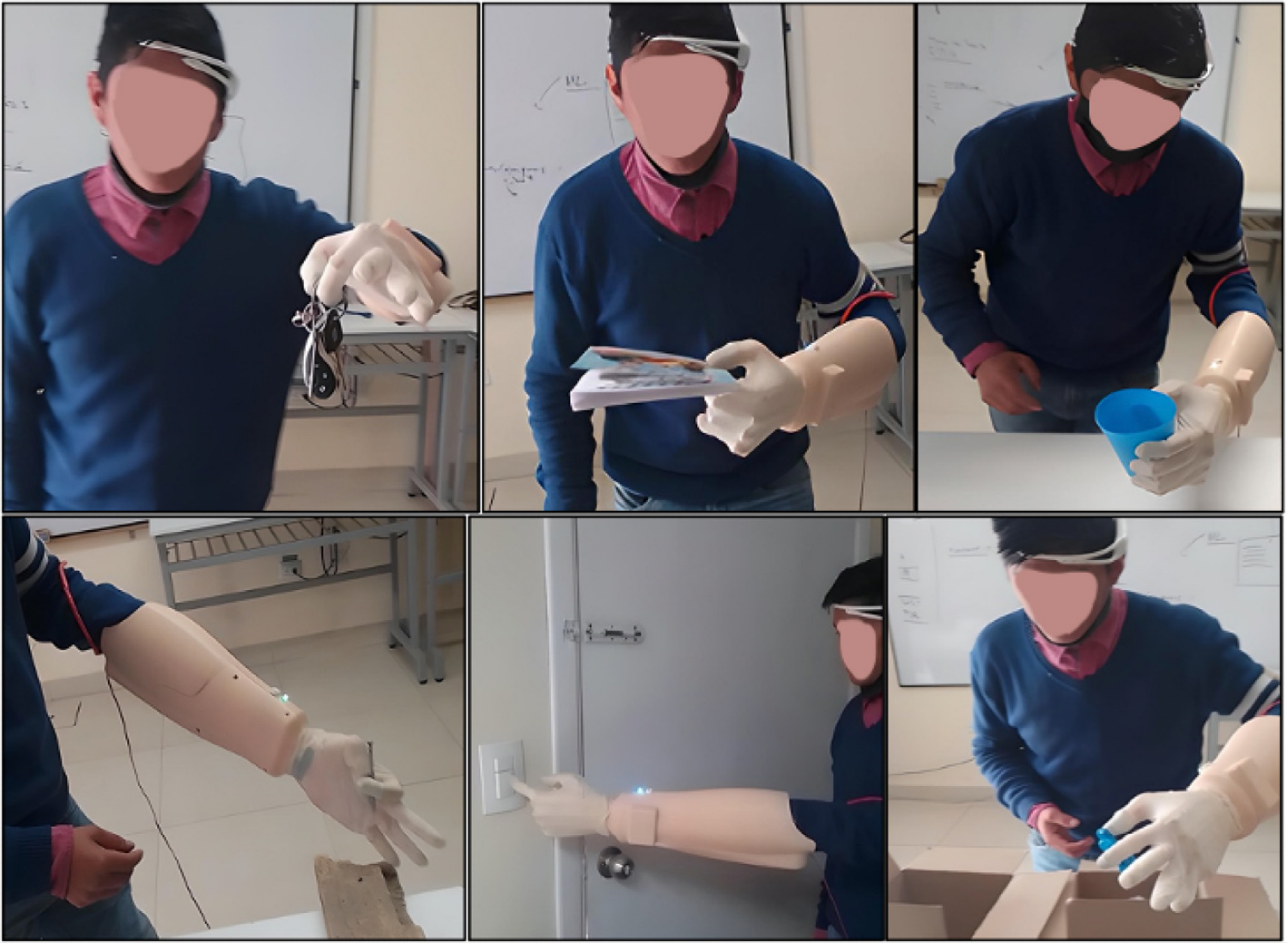
Testing of the prosthesis in use.

Fig. 25 shows the prosthesis successfully performing grips with different objects.

**Fig. 26:**
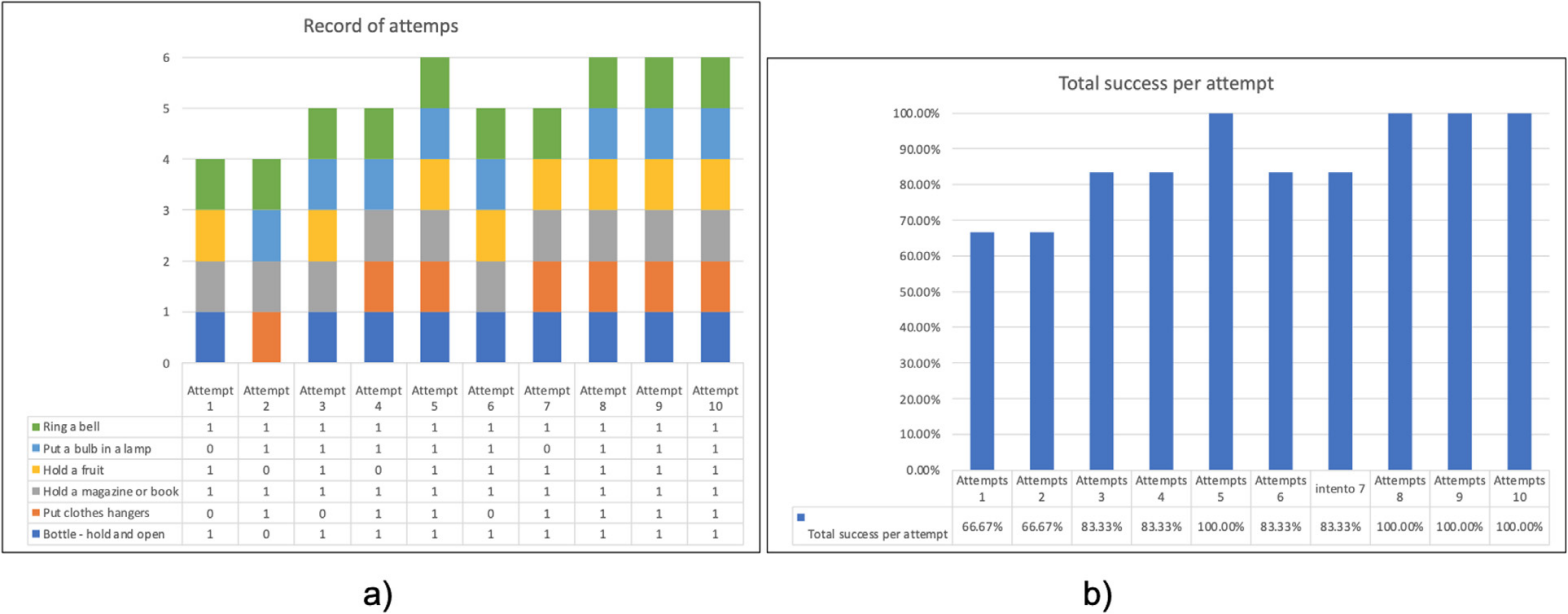
(a) Graph of the ten-attempt test, (b) Percentage of success per attempt.

To validate this prosthesis, ten attempts were made to execute tasks set by the test person. The results are shown graphically in Fig. 26(a), where the tasks that were correctly performed in each attempt are observed. Fig. 26(b) shows the percentage of correctness for each attempt. We conclude from the graph that the percentage correct is greater than 60% and increases as the attempts increase. This is due to the user’s dexterity and ability to adapt to the use of the prosthesis. We can predict that, as the prosthesis is used, the manipulation and the success rate will improve.

**Fig. 27:**
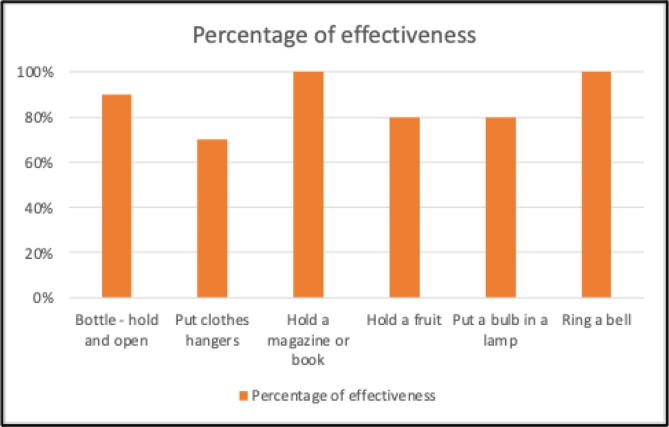
Percentage of success for each activity or task performed.

Fig. 27 shows the percentage of success for each activity. We observe that the most difficult task is to place a clothes hanger, due to the precision required to hold the small hook. Finally, we concluded that the average success rate is 86.67%, which is quite acceptable compared to commercial prostheses.

### 7.2 Apparent haptic system

**Fig. 28:**
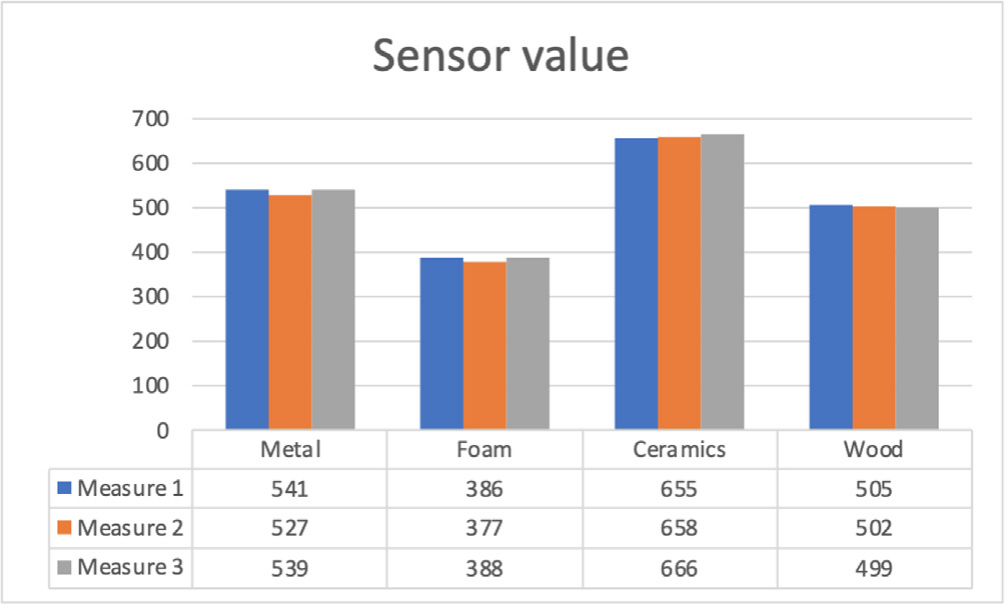
Values obtained with the force sensor when touching different objects.

In the tests when touching different materials, a variation in the reading of the force sensor is observed according to Fig. 28.

### 7.3 Object recognition with Machine learning

**Fig. 29:**
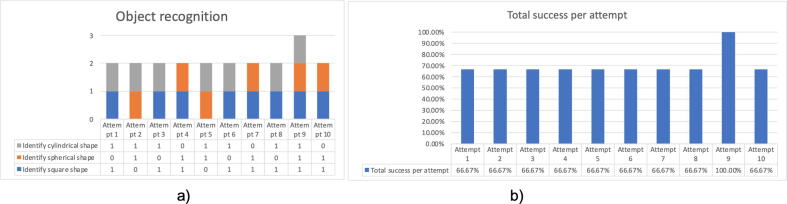
(a) Graph of the ten-attempt test, (b) Percentage of success per attempt.

To validate object recognition, ten attempts were made to recognize cubic, spherical and cylindrical objects; the results are shown in Fig. 29.

**Fig. 30:**
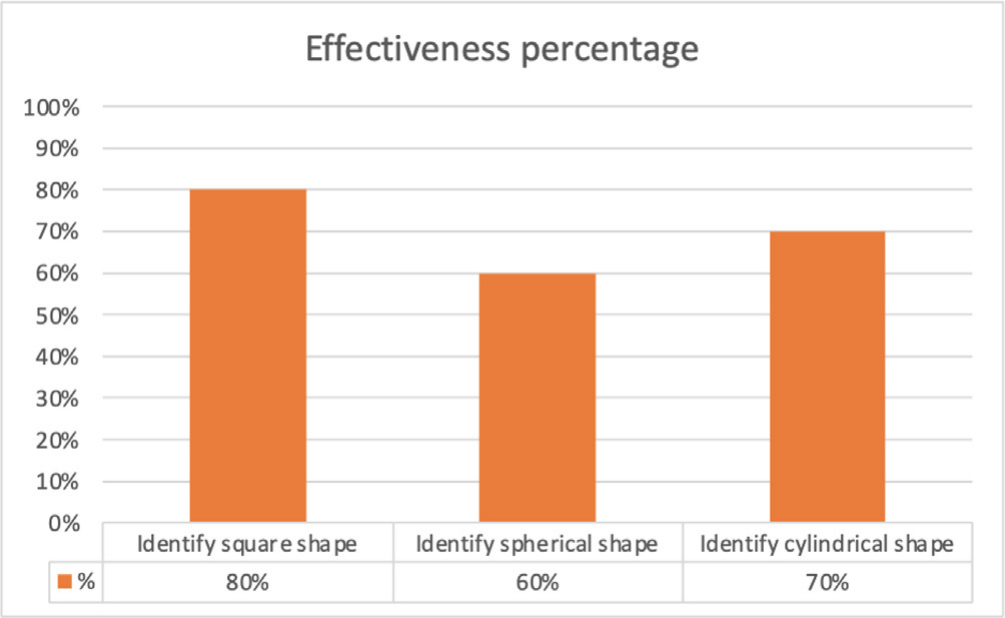
Percentage of success for each shape to be identified.

Fig. 30 shows the percentage of success in recognizing objects. We observe that the easiest shape to recognize is the cubic shape and the most difficult is the spherical shape. At the end of the tests, it was concluded that the prosthesis has an average effectiveness of 70% in recognizing objects.

## 8 Summary of Limitations of the Prosthesis

While our recent robotic hand and arm prosthesis provides superior functionality and high success rates in terms of accuracy, it is important to address certain inherent limitations in its design and use. These limitations are not specific to our prosthesis alone but are common to robotic prosthetics in general.

1. **Bluetooth Communication Range**: The prosthesis communicates with the controlling device via Bluetooth technology, which has a range of approximately 10 meters. Therefore, the prosthesis may lose functionality if the user moves beyond this distance from the control device.
2. **Battery Life**: The prosthesis has a battery life of about 5 h on a single charge. While this might be sufficient for day-to-day tasks, it might prove insufficient for users requiring prolonged use. Using batteries with greater capacity could extend this life, but it could increase the weight of the prosthesis.
3. **Water Resistance**: Our prosthesis is not water-resistant. Submerging it in water could damage the electronic components, resulting in a loss of functionality.
4. **Limitations of 3D Printed Prosthetics**: While 3D printing has allowed for the rapid and customized production of prosthetics, these may present limitations in terms of strength and durability. 3D printed prosthetics might not be as robust as those made through traditional methods, and therefore might require more frequent replacements.
5. **Adaptation and Comfort**: Adjusting to the prosthesis can take time and users may experience initial discomfort.
6. **Complexity of Fine Movements**: While our prosthesis is capable of a wide range of movements, fine and precise movements may still be a challenge. Difficulty in performing tasks that require a high degree of dexterity may be a limitation for some users.

We anticipate that the recognition and ongoing surmounting of these limitations will allow us to develop more efficient and comfortable prosthetics in the future.

## 9 Conclusion

Zero Arm is a low-cost prosthesis that can mimic basic hand movements and perform the same grips as expensive robotic prosthetics on the market. It has four degrees of freedom and is controlled by electroencephalographic signals. In addition, it has a wireless communication module that allows it to be controlled from a smartphone using an application.

In this work, the construction process of the prosthesis hardware is described and shared, from 3D printed mechanical components to an electronic printed circuit board, including all the necessary code for the optimal functioning of the system. The prosthesis was experimentally validated through tests similar to those carried out at Cybathlon, where the most modern and expensive prostheses compete.

The total cost of the Zero Arm is approximately USD $600, making it affordable compared to prosthetics on the international market. Its portability, flexibility, affordability, and practicality make it attractive to be replicated.

## Ethics statements

The work described has been carried out in accordance with The Code of Ethics of the World Medical Association (Declaration of Helsinki) for experiments involving humans. In addition, informed consent was obtained from humans for the validation of the prosthesis.

## CRediT authorship contribution statement

**Diego Ronaldo Cutipa-Puma:** Software, Visualization, Investigation, Writing - original draft. **Cristian Giovanni Coaguila-Quispe:** Conceptualization, Methodology, Investigation, Validation, Formal analysis, Supervision. **Pablo Raul Yanyachi:** Writing - review & editing.

## Declaration of Competing Interest

The authors declare that they have no known competing financial interests or personal relationships that could have appeared to influence the work reported in this paper.
